# Public Awareness and Perceptions of the 988 Suicide & Crisis Lifeline Among California Adults

**DOI:** 10.1007/s10597-026-01615-8

**Published:** 2026-04-28

**Authors:** Amanda Aubel, Julia Lund, Garen Wintemute, Nicole Kravitz-Wirtz

**Affiliations:** Centers for Violence Prevention, Department of Emergency Medicine, University of California Davis, Sacramento, United States

**Keywords:** Suicide Prevention, Safety, Mental Health, Crisis Services, Public Health, Inequity

## Abstract

Suicide is a major public health issue that disproportionately affects certain populations. The 988 Suicide & Crisis Lifeline (988) was established in July 2022 as an accessible, easy-to-remember resource for individuals in crisis. Maximizing its impact relies on public knowledge and willingness to use the service. Using data from a cross-sectional survey of California adults (*n* = 3531), fielded online in November 2024, this study assessed awareness and likelihood of using 988, and barriers and facilitators to use, overall and among groups known to be at increased risk for suicide. One in 4 California adults (26.5%; 95% CI: 24.4–28.9) had heard of 988. After reading a brief description of the service, 71.0% (95% CI: 69.1–73.8) said they would be somewhat or very likely to contact 988 if they or someone they knew were experiencing a crisis. Awareness was higher among younger adults, but likelihood of use was lower than older adults. Black adults were more often aware of and likely to use 988 than other racial/ethnic groups. Similar percentages of gun owners and non-owners were likely to use 988. Respondents identified the following factors as most important to their decision to contact 988: immediate access to a crisis counselor, no-cost services, dispatch of mental health professionals in cases of imminent risk, and assurance that the person in crisis would not be taken to jail. Findings provide insight into 988’s implementation and reach in California, which can inform strategies for enhancing effectiveness and equitable access and service use in times of crisis.

**Supplementary Information:**

The online version contains supplementary material available at https://doi.org/10.1007/s10597-026-01615-8.

## Introduction

Suicide is a major public health problem in the United States (US), claiming nearly 50,000 lives each year (Centers for Disease Control and Prevention, National Center for Health Statistics, n.d.). Although suicide affects individuals across all demographics, some populations face higher risk. Men aged 75 and older have one of the highest suicide rates (40.7 per 100,000 in 2023). Although rates are lower among youth and young adults, suicide remains one of the top three leading causes of death among those aged 10–24, and the 62% increase in rate between 2007 and 2021 in this age group (from 6.8 to 11.0) has far outpaced the 26% increase in the overall suicide rate (Centers for Disease Control and Prevention, National Center for Health Statistics, n.d.; Curtin & Garnett, 2023). Suicide patterns reflect not only age differences but also broader period and birth cohort effects, with substantial heterogeneity by sex, race, and suicide method (Martínez-Alés et al., 2021; Phillips, 2014).

Individuals who identify as lesbian, gay, bisexual, transgender, or another sexual or gender minority (LGBT+) are also at elevated risk. In 2021, more than a quarter of lesbian, gay, and bisexual high school students reported attempting suicide in the past year – five times the rate among heterosexual students – and 40% of transgender adults reported having made a suicide attempt at some point in their lifetime compared with 2.4% of the general population (Jones et al., 2022; Rastogi et al., 2025). Racial and ethnic inequities are also stark, with American Indian and Alaska Native (AI/AN) people experiencing the highest suicide rate of any group (28.1 per 100,000 in 2021), and rates rising significantly between 2018 and 2021 among Hispanic (7% increase), AI/AN (26% increase), and Black (19% increase) individuals – especially Black youth and young adults – while declining among White people (4% decrease) (D. M. Stone et al., 2023).

These disparities stem from the inequitable distribution of structural, socioeconomic, and situational conditions, including poverty, geography, and access to lethal means, which influence suicide risk. Economic and financial strain are associated with increased suicide at both the individual and population levels (Stack, 2021), whereas even modest increases in state minimum wages have been linked to reductions in suicide, particularly among individuals with lower educational attainment and during periods of high unemployment (Gertner et al., 2019; Kaufman et al., 2020). Suicide rates tend to increase as population density decreases, with urban-rural differences widening over time (Pettrone & Curtin, 2020). Access to firearms is another critical factor: firearms are the most common and lethal method of suicide (accounting for > 50% of deaths) (Centers for Disease Control and Prevention, National Center for Health Statistics, n.d.), and research in California suggests that handgun ownership increases the risk of firearm suicide by nearly 8 times for men and 35 times for women (Studdert et al., 2020). It is therefore plausible that higher rates of gun ownership among veterans contribute to their elevated suicide risk: in 2022, age-adjusted suicide rates were 92% higher for female veterans and 44% higher for male veterans than comparable non-veteran US adults (US Department of Veterans Affairs, Office of Suicide Prevention, 2024).

In response to the urgent need for effective and comprehensive suicide prevention strategies, the US government established and launched the 988 Suicide and Crisis Lifeline (hereafter “988”) in July 2022. This initiative transitioned the National Suicide Prevention Lifeline, which had operated under a ten-digit number since 2005, to an accessible, easy-to-remember three-digit dialing code linked to a coordinated network of over 200 crisis centers nationwide (NAMI North Carolina, 2024). Designed to enhance service quality and expand access to timely and appropriate care, the transition to 988 aimed to expand the pool of users beyond individuals experiencing acute suicidal ideation while also providing a non-law enforcement alternative to calling 911 for needs related to broader psychological and emotional crises. Trained crisis counselors provide free and confidential call, text, and chat support 24/7 and are required to initiate emergency interventions when help-seekers are determined to be at “imminent risk” and attempts to de-escalate or collaborate on less invasive alternatives have failed (Vibrant Emotional Health, 2022). While infrequent, such emergency interventions, which vary by jurisdiction and can include sending law enforcement, emergency medical services, or mobile crisis teams to a help-seeker’s location, can occur with or without the help-seeker’s consent (Drapeau, 2024).

Following the launch of the new three-digit number, call volume to the Suicide & Crisis Lifeline increased nationally and in every state, though to varying degrees (Purtle et al., 2023b). In its first two years, 988 received more than 10 million calls, texts, and chats (NAMI North Carolina, 2024). While promising, publicly available performance metrics provide limited insight into the public’s perceptions of and experiences with 988 (Substance Abuse and Mental Health Services Administration, 2023; 988 Lifeline, n.d.), and empirical research on the topic remains scarce (Purtle & Lindsey, 2025). Nationally representative surveys have yielded varying estimates of public awareness of 988. In April 2023, a Pew survey found that 18% of US adults had heard of “the new 3-digit line 988” (Velázquez, 2023). Two months later, Purtle and colleagues found that, when provided with a description of 988 upfront, 42% of US adults reported having heard of the service (Purtle et al. 2023a). While less than 1% of the general population said they had used 988 for themselves, respondents with “serious” past 30-day psychological distress were significantly more likely than those with no distress to have heard of 988 (47% vs. 40%) and to have used it for themselves (6% vs. 0.2%), suggesting that the service is reaching those with the greatest need, in line with its intended purpose (Purtle et al. 2023a).

However, much remains to be learned about the prevalence of 988 use, its perceived acceptability, and the factors that may facilitate or hinder engagement. In the Purtle et al. survey, respondents with “serious” distress, compared to those with no distress, less frequently said they were “very likely” to use 988 in the future if they or a loved one were experiencing a mental health crisis or suicidality (22% vs. 26%) (Purtle et al. 2023a). More generally, Pew reported that 35% of US adults were “highly likely” and 37% were “somewhat likely” to contact 988 if they or someone they knew “needed help” (Velázquez, 2023). To our knowledge, no peer-reviewed studies have examined why individuals may be more or less willing to contact 988. However, several publicly available reports offer valuable insights, including some based on qualitative research with people disproportionately impacted by suicide and those with lived experiences of mental health crises (Ad Council Research Institute, 2023; Leigh et al., 2024; National Alliance on Mental Illness (NAMI), 2024; Velázquez, 2023). Nationally, about two in five adults report being concerned that contacting 988 might result in a law enforcement response, being forced to go to the hospital, being charged for services they could not pay, or other people finding out they had called (Velázquez, 2023). These concerns, among others, may be heightened among people with mental health issues and marginalized social identities – including Black, Brown, LGBT+, and disabled individuals – who are particularly vulnerable to the traumatic consequences that can result from non-consensual emergency interventions that may involve law enforcement, involuntary psychiatric hospitalization, incarceration, or other potentially harmful experiences (Ad Council Research Institute, 2023; Leigh et al., 2024; Velázquez, 2023). On the other hand, the provision of immediate and 24/7 access to a crisis counselor appears to be among the top factors encouraging individuals to contact 988 (Ad Council Research Institute, 2023; National Alliance on Mental Illness (NAMI), 2024).

Given that 988 and crisis care systems operate primarily at the state and local levels, state-specific data are essential for evaluating implementation and impact; however, the availability of such data remains limited. To address this gap, this study investigates Californians’ awareness of and likelihood to use 988, and the factors that may facilitate or deter use, in a general population sample and among subgroups at elevated risk for suicide, including LGBT+ individuals, veterans, gun owners, and low-income individuals.

## Methods

### Sample

Data for this study come from the 2024 California Safety and Wellbeing Survey (CSaWS 2024), the third wave of a repeated statewide cross-sectional survey designed by researchers at the University of California, Davis (UC Davis) Centers for Violence Prevention and administered by the survey research firm Ipsos. Each wave of the survey is designed to address pressing policy and public health issues related to safety and violence, with an emphasis on gun violence. CSaWS 2024 was administered online in English and Spanish from November 4–25, 2024. Survey respondents were drawn from the Ipsos KnowledgePanel, an online research panel widely used in health and safety research in the US. To establish a nationally representative panel, members are randomly recruited on an ongoing basis through address-based probability sampling using data from the US Postal Service’s Delivery Sequence File (Ipsos, n.d.). A probability-proportional-to-size procedure was used to select a study-specific sample. All members who were 18 years and older and non-institutionalized residents of California households were eligible to participate in CSaWS 2024. Invitations were sent by e-mail; automatic reminders were e-mailed three days later and periodically thereafter. Of 5,841 invitations, 3,631 individuals completed the survey (completion rate, 62.2%); 100 cases were excluded from analysis for refusing more than half of the survey items (*n* = 13), providing an invalid or non-California zip code (*n* = 49), or refusing to provide a zip code (*n* = 38), resulting in a final sample of 3,531 respondents. Informed consent language was provided at the beginning of the survey and study initiation constituted consent. The median survey completion time was 23 min. This study follows the American Association for Public Opinion Research (AAPOR) reporting guidelines and was approved by the University of California, Davis Institutional Review Board.

### Measures

Respondents were asked whether they had “heard of the 3-digit telephone line, 988?” (yes/no). Next, they read a brief description of 988 and were asked, “How unlikely or likely would you be to contact 988 if you or someone you know was experiencing a suicidal, substance use, or mental health crisis, or was in emotional distress?” Response options were very unlikely, somewhat unlikely, somewhat likely, and very likely. A convenience subsample of respondents (*n* = 250) was shown a list of 10 factors that might influence their decision to contact 988 and asked, “When considering whether to contact 988, how important are the following things to you?” Response options were not at all important, somewhat important, and very important. Full-text survey items are in the Appendix, and sociodemographic characteristics of the subsample can be found in Appendix Table 1.

We measured respondents’ gun ownership status with two questions: “Do you or does anyone you live with currently own any type of gun?” and if yes, “Do you personally own a gun?” Respondents were then classified as gun owners, non-owners who live with gun owners, or as having no guns in the home. Additional sociodemographic characteristics, including respondents’ sex, age, household income, residential location, and race and ethnicity, were collected as part of initial KnowledgePanel membership. Race and ethnicity were jointly categorized using responses to two questions: “Are you Spanish, Hispanic, or Latino?” (hereafter, Latine) and then, “Please indicate what you consider your race to be” from one or more of the following US Census Bureau categories: White, Black or African American, American Indian or Alaska Native (AI/AN), Asian, and Native Hawaiian or Pacific Islander. Respondents who did not endorse Spanish, Hispanic, or Latino ethnicity and who identified as AI/AN (*n* = 10), Native Hawaiian or other Pacific Islander (*n* = 5), or more than one race (*n* = 86) were combined into an “other/multi-race” category to maintain sufficient sample size for analysis.

Information on sexual orientation, gender identity, and veteran status was obtained from supplemental KnowledgePanel surveys and merged with the CSaWS 2024 dataset by Ipsos. We classified respondents as LGBT + if they identified as lesbian, gay, bisexual, transgender, non-binary, or some “other” sexual orientation or gender identity; those who identified as straight (heterosexual) and cisgender were classified as non-LGBT+. Veterans were identified as respondents who answered ‘yes’ to the question, “Did you ever serve on active duty in the US Armed Forces?” Approximately 10% of respondents were missing data for these measures because they had not yet completed the supplemental survey; an additional 1–2% refused to answer each of these questions.

### Analysis

Responses were weighted to be statistically representative of the non-institutionalized adult population of California, as reflected in the 2018–2022 American Community Survey. The final survey weight, provided by Ipsos, combined a design weight, which adjusts for the initial probability of selection into KnowledgePanel, with a study-specific post-stratification weight that accounts for differential survey nonresponse using an iterative proportional fitting (raking) procedure to align the weighted sample with benchmark distributions of age, sex, race/ethnicity, education, household income, language proficiency, and geographic region among California adults (Ipsos, n.d.). We calculated weighted percentages and 95% confidence intervals (CI) for each measure or cross-tabulation of measures using the survey and weighting commands in Stata statistical software version 19.5 (StataCorp).

To contextualize the regional estimates from the survey, we also computed the average annual suicide rates in each of California’s 10 Census regions, using publicly available public health data and a single imputation technique adapted from Erdman et al. (2021) for suppressed small numbers. Additional details about these calculations can be found in the Appendix. These analyses and the corresponding maps were completed in R version 4.1.2 (R Core Team 2020).

## Results

Of 3,531 respondents (mean [SD] age, 48.5 [17.3] years), 51.8% were female (95% CI: 49.3–54.3) and 39.7% (95% CI: 37.3–42.1) identified as White, 35.9% (95% CI: 33.5–38.4) as Latine, 15.4% (95% CI: 13.5–17.5) as Asian, 5.2% (95% CI: 4.2–6.5) as Black, and 3.8% (95% CI: 2.9–5.0.9.0) as other/multi-race (Table 1). One-fifth of the sample (21.8%; 95% CI: 19.9–23.7) had a household income below $50,000, 11.2% (95% CI: 9.6–13.0) identified as LGBT+, and 4.9% (95% CI: 4.1–5.8) were veterans. More than one in seven respondents (15.4%; 95% CI: 13.8–17.2) were gun owners, and an additional 11.3% (95% CI: 9.6–13.1) were non-owners who live with owners.

**Table 1:** Sociodemographic characteristics of survey respondents (*n*=3531)

	Weighted % (95% CI)	Unweighted N
Sex		
Female	51.8 (49.3–54.3)	1943
Male	48.2 (45.7–50.7)	1943
Age		
18–29	14.6 (12.6–17.0)	213
30–44	31.0 (28.6–33.6)	673
45–59	25.4 (23.4–27.6)	891
60+	28.9 (27.0–30.9)	1754
Race/ethnicity		
Asian	15.4 (13.5–17.5)	314
Black	5.2 (4.2–6.5)	215
Latine	35.9 (33.5–38.4)	1118
White	39.7 (37.3–42.1)	1783
Other/multi-race	3.8 (2.9–5.0)	101
Household income		
Less than $50,000	21.8 (19.9–23.7)	1161
$50,000 to $99,999	25.9 (23.7–28.1)	1013
$100,000 to $149,999	19.6 (17.6–21.7)	587
$150,000 or more	32.8 (30.4–35.3)	770
Region		
Central Coast	6.0 (5.0–7.2)	242
Inland Empire	10.8 (9.3–12.4)	419
Los Angeles County	26.1 (23.9–28.5)	826
North Coast	2.7 (2.1–3.5)	109
Northern San Joaquin Valley	4.6 (3.7–5.7)	175
Orange County	8.2 (6.9–9.7)	237
San Diego - Imperial	8.6 (7.3–10.0)	326
San Francisco Bay Area	18.7 (16.8–20.8)	606
Southern San Joaquin Valley	5.9 (4.8–7.0)	264
Superior California	8.5 (7.2–10.0)	327
Gun ownership status		
Gun owner	15.4 (13.8–17.2)	618
Non-owner who lives with owner	11.3 (9.6–13.1)	333
No guns in home	67.0 (64.5–69.3)	2399
Don’t know	5.5 (4.3–6.9)	162
Refused	0.9 (0.5–1.6)	19
LGBT+		
Yes^a^	11.2 (9.6–13.0)	329
No (Heterosexual, cisgender)	76.2 (73.8–78.4)	2797
Not asked	10.8 (9.3–12.5)	365
Refused	1.8 (1.2–2.8)	40
Veteran		
Yes	4.9 (4.1–5.8)	270
No	84.3 (82.4–86.0)	2897
Not asked	10.3 (8.8–12.0)	349
Refused	0.5 (0.3–1.0)	15

^a^LGBT+ includes respondents who identified as lesbian, gay, bisexual, transgender, non-binary, or some “other” sexual orientation or gender identity

Approximately one-quarter (26.5%; 95% CI: 24.4–28.9) of respondents said they had heard of 988 (Table 2). Awareness was highest among respondents aged 18–29 years (36.4%; 95% CI: 28.8–44.8), those who identified as Black (35.0%; 95% CI: 24.6–47.1) or other/multi-race (46.5%; 95% CI: 32.7–60.9), and LGBT+ respondents (35.5%; 95% CI: 28.1–43.7). Veterans (29.6%: 95% CI: 21.8–38.8), respondents with household incomes of $150,000 or more (30.0%; 95% CI: 25.8–34.5), and non-gun owners living with owners (30.4%; 95% CI: 23.3–38.5) were slightly more likely to have heard of 988 than their comparative subgroups. Levels of awareness were similar among male and female respondents, and were lowest among Asian respondents (20.2%; 95% CI: 14.9–26.7) and those aged 60 years and older (20.3%; 95% CI: 17.9–23.0).

**Table 2:** Awareness of 988, overall and by sociodemographic characteristics (n=3531)

	Yes % (95% CI) [*n*]	No % (95% CI) [*n*]
Total	26.5 (24.4–28.9) [882]	72.7 (70.4–74.9) [2633]
Sex		
Female	26.9 (24.0–30.1) [496]	72.3 (69.1–75.3) [1440]
Male	26.1 (22.9–29.6) [386]	73.1 (69.7–76.4) [1193]
Age		
18–29	36.4 (28.8–44.8) [74]	62.4 (54.0–70.2) [136]
30–44	28.2 (24.0–32.9) [204]	70.8 (66.0–75.1) [462]
45–59	25.9 (22.1–30.2) [229]	73.4 (69.1–77.4) [659]
60+	20.3 (17.9–23.0) [375]	79.4 (76.7–81.9) [1376]
Race/ethnicity		
Asian	20.2 (14.9–26.7) [56]	79.8 (73.3–85.1) [258]
Black	35.0 (24.6–47.1) [59]	64.7 (52.7–75.1) [155]
Latine	26.9 (23.1–31.0) [281]	72.0 (67.9–75.8) [828]
White	25.7 (22.7–28.8) [446]	73.5 (70.3–76.5) [1331]
Other/multi-race	46.5 (32.7–60.9) [40]	53.5 (39.2–67.3) [61]
Household income		
Less than $50,000	25.9 (21.8–30.5) [277]	73.6 (68.9–77.7) [877]
$50,000 to $99,999	25.9 (21.7–30.6) [236]	72.8 (68.0–77.1) [772]
$100,000 to $149,999	22.4 (18.3–27.1) [154]	77.0 (72.2–81.2) [432]
$150,000 or more	30.0 (25.8–34.5) [215]	69.6 (65.0–73.8) [552]
Gun ownership status		
Gun owner	28.5 (23.2–34.4) [150]	71.5 (65.6–76.8) [467]
Non-owner who lives with owner	30.4 (23.3–38.5) [92]	69.7 (61.5–76.7) [241]
No guns in home	26.1 (23.6–28.9) [604]	73.2 (70.4–75.8) [1786]
LGBT+		
Yes^a^	35.5 (28.1–43.7) [108]	64.4 (56.2–71.9) [220]
No (Heterosexual, cisgender)	25.6 (23.2–28.2) [678]	74.0 (71.5–76.5) [2113]
Veteran		
Yes	29.6 (21.8–38.8) [74]	70.2 (61.0–78.0) [194]
No	26.6 (24.2–29.1) [722]	73.0 (70.4–75.4) [2168]

^a^LGBT+ includes respondents who identified as lesbian, gay, bisexual, transgender, non–binary, or some “other” sexual orientation or gender identity

Row percentages may not sum to 100% because refusals are not shown

**Fig. 1:**
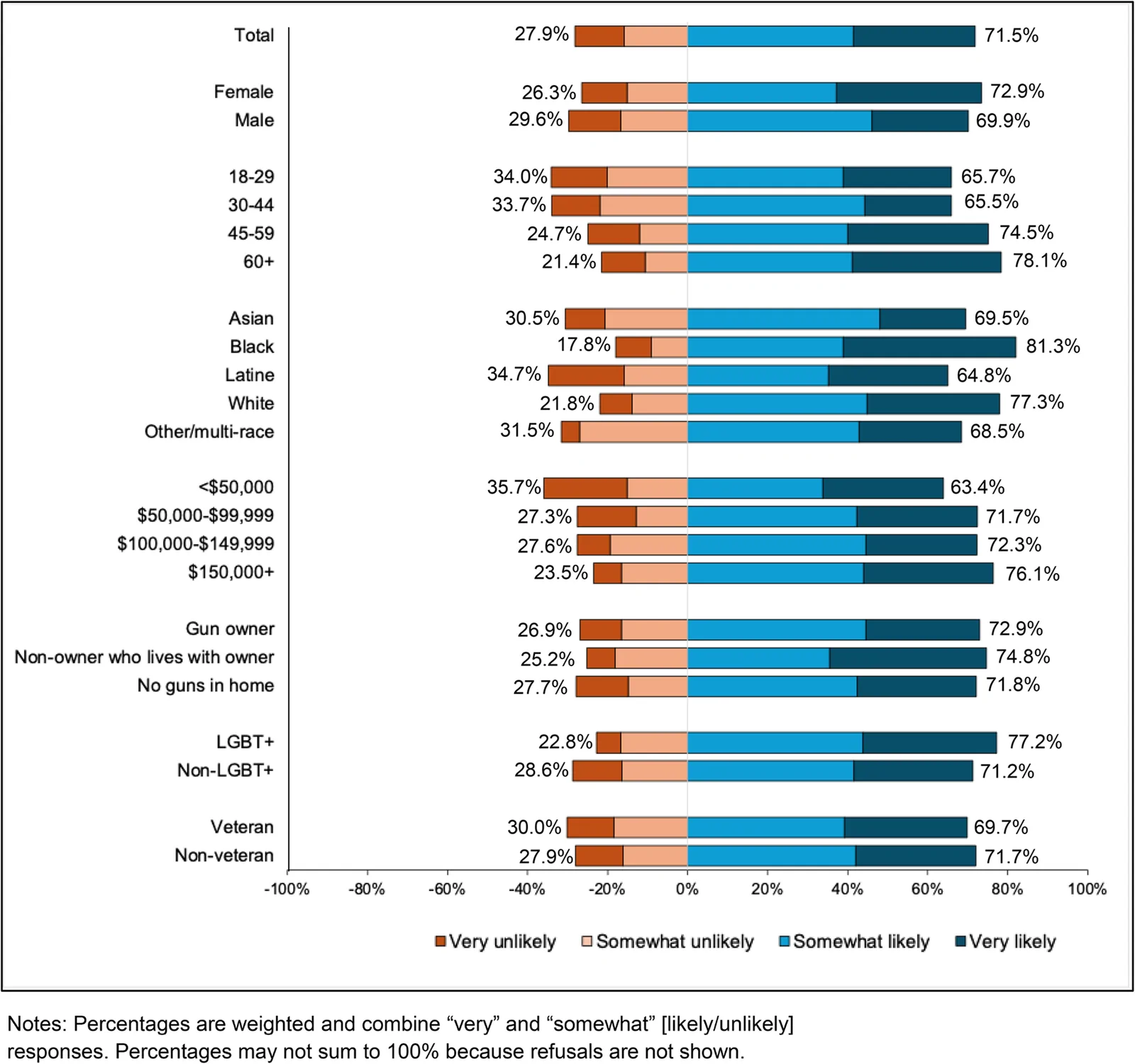
Likelihood of contacting 988, overall and by sociodemographic characteristics (*n* = 3531)

After receiving a brief description of 988, 71.5% (95% CI: 69.1–73.8) of respondents said they would be somewhat (41.3%; 95% CI: 38.8–43.7) or very (30.2%; 95% CI: 28.0–32.5.0.5) likely to contact 988 if they or someone they know was experiencing a crisis (Fig. 1 and Appendix Table 2). Likelihood was highest (> 75% reporting somewhat or very likely) among respondents aged 60 years and older (78.1%; 95% CI: 74.9–80.9), those who identified as Black (81.3%; 95% CI: 72.7–87.7) or White (77.3%; 95% CI: 74.0–80.3.0.3), those with household incomes of $150,000 or more (76.1%; 95% CI: 71.7–80.0), and LGBT+ respondents (77.2%; 95% CI: 70.4–82.9). Levels of likelihood were similar and remained high (≥ 70% somewhat/very likely) across sex, gun ownership status, and veteran status. Of all subgroups, respondents with household incomes below $50,000 most often reported being very unlikely to contact 988 (20.7%; 95% CI: 16.8–25.2).

**Fig. 2:**
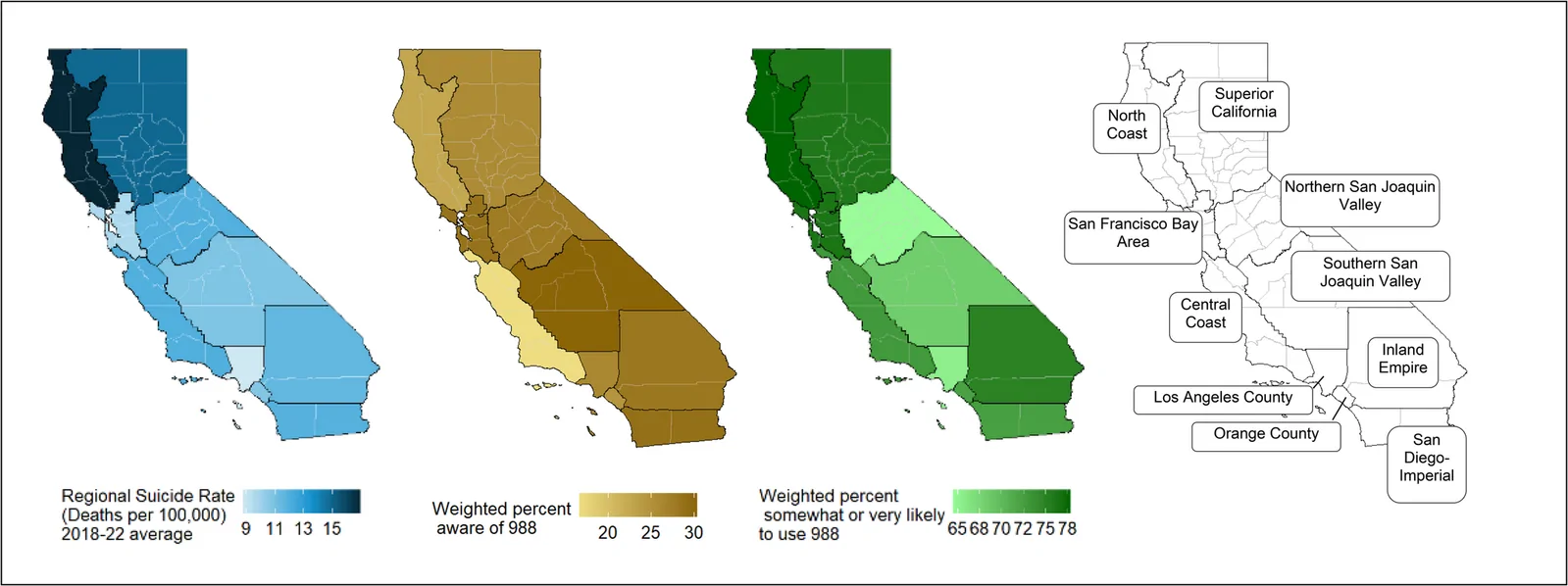
Suicide rates and awareness and likelihood of using 988, by California region

Awareness and likelihood also varied across geographic regions (Fig. 2 and Appendix Table 3). Awareness of 988 was highest in the Southern San Joaquin Valley (30.3%; 95% CI: 22.8–39.1) and lowest in the Central Coast (16.6%; 95% CI: 11.4–23.6). Likelihood of use ranged from 64.5% (95% CI: 53.2–74.4) to 77.8% (95% CI: 62.5–88.1), in the Northern San Joaquin Valley and North Coast region, respectively. The regions with the highest average annual suicide rates – the North Coast (16.9 per 100,000) and Superior California (14.9 per 100,000) – tended to have relatively lower levels of 988 awareness (< 26%) but among the highest likelihood of use (> 75%).

**Fig. 3:**
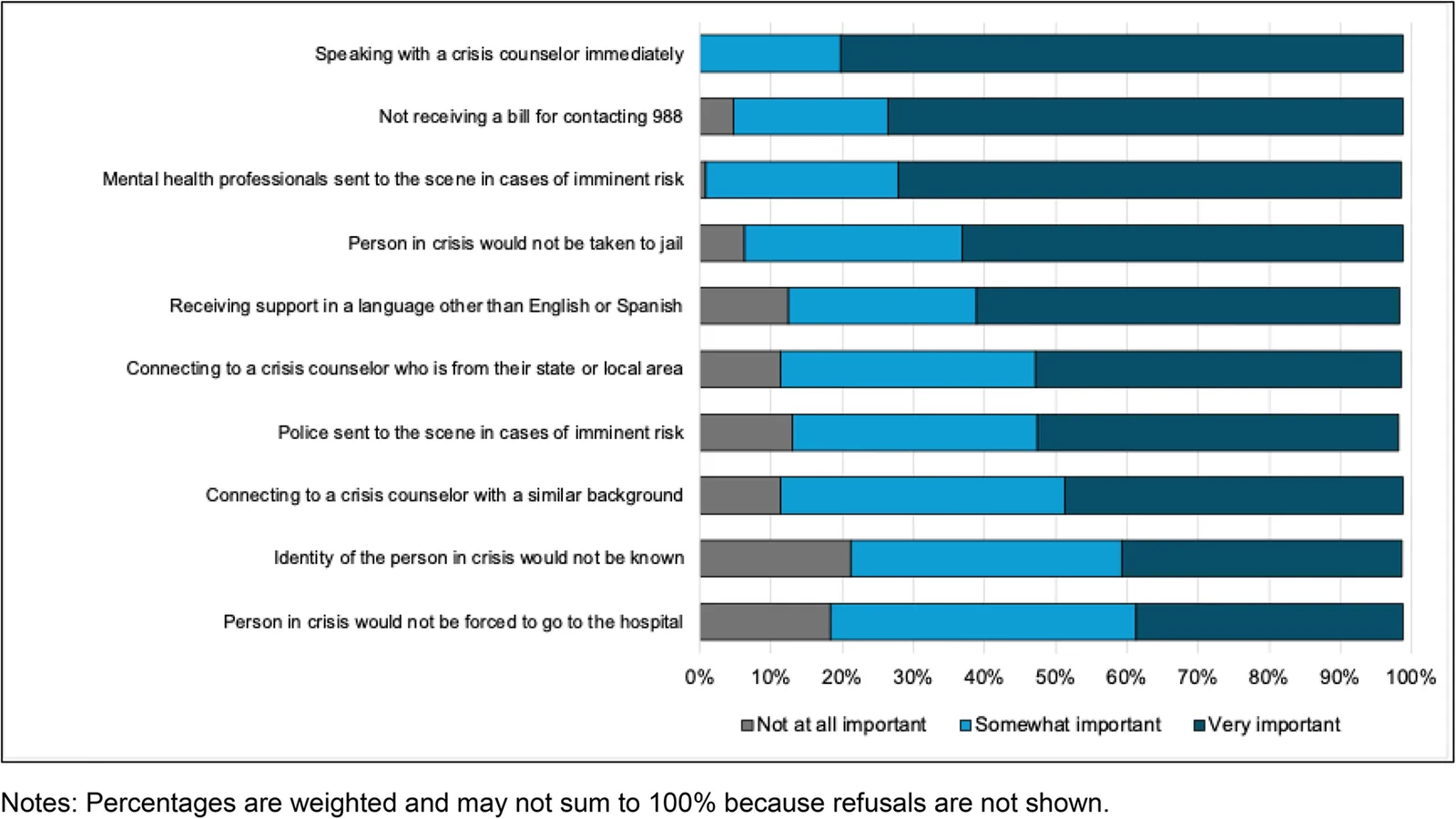
Importance of factors in decision to contact 988 (*n* = 250)

When presented with a list of potential barriers or facilitators to contacting 988, a majority of respondents (77–99%, depending on the item) rated each factor as at least somewhat important in their decision (Fig. 3 and Appendix Table 4). Knowing that the person in crisis would be able to speak with a crisis counselor immediately (79.0%, 95% CI: 69.6–86.1), that they would not receive a bill for contacting 988 (72.3%; 95% CI: 63.1–79.9), and that mental health professionals would be sent to the scene in cases of imminent risk (70.7%; 95% CI: 60.9–78.9) were most frequently rated as very important. Approximately three in five respondents said knowing that the person in crisis would not be taken to jail (61.8%; 95% CI: 52.1–70.7) and being able to receive support in a language other than English or Spanish (59.5%; 95% CI: 50.0–68.3.0.3) were very important. Conversely, a plurality of respondents indicated that it was not at all important to their decision to contact 988 to know that the identity of the person in crisis would not be known to the person answering the call (21.3%; 95% CI: 14.3–30.4) or to know that the person in crisis would not be forced to go to the hospital (18.4%, 95% CI: 11.9–27.4). Subgroup analyses can be found in Appendix Tables 5-14.

## Discussion

This general population survey study adds to a small but growing body of literature on public awareness and perceptions of the 988 Suicide and Crisis Lifeline – an area where peer-reviewed evidence remains limited – with an emphasis on groups at increased suicide risk. Our findings provide critical insights into the implementation and reach of 988 in California, which can inform strategies for enhancing effectiveness and equitable access to the resource in times of crisis. We found that, more than two years after its launch, only one in four California adults reported being aware of 988. Although this exceeds the most comparable national estimate (18%) from nine months after 988’s launch (Velázquez, 2023), this finding highlights the need for continued public education and outreach to make 988 a universally recognized resource, akin to 911, and to maximize its utilization and impact.

Although awareness of 988 was generally low, reported likelihood of use was high across sociodemographic groups. Support for 988 may stem from its positioning as an alternative to 911 for mental and other behavioral health crises. National survey data indicate that Americans across subgroups feel more comfortable seeking help from a 988 crisis counselor during a mental health crisis or emergency than from a 911 operator or law enforcement officer (National Alliance on Mental Illness (NAMI), 2024). These findings underscore the growing support for alternative 911 crisis response models that deploy behavioral health professionals – with or without law enforcement – to non-life threatening situations. This approach, backed by a majority of California voters, reflets a broader shift toward public health-oriented approaches that prioritize unarmed, community-based interventionists over traditional law enforcement responses (Public Health Advocates, 2023; Spolum et al., 2023). While such models represent a critical evolution in local crisis intervention ecosystems, they have yet to be widely implemented due to a lack of public funds, alongside challenges related to staff availability and training, and support for integration across systems.

Non-policing alternatives, including 988, may be especially important for marginalized and minoritized communities disproportionately harmed by law enforcement-led responses to behavioral health-related calls for service (GenForward & Movement for Black Lives, 2023; Spolum et al., 2023). In our study, Black respondents more often indicated that knowing police would be dispatched in cases of imminent risk and that the person in crisis would not be taken to jail were “very important” considerations when deciding whether to contact 988 (Appendix Tables 8 and 13). These findings align with national data showing that Black Americans are most likely to report feeling unsafe calling 911 for a mental health crisis (59%) and afraid that police might harm them or their loved one during such a response (77%) (National Alliance on Mental Illness (NAMI), 2024).

Although concerns about police involvement may deter some individuals from calling 988, our findings suggest that the potential for workers trained in mental health to be sent to the scene may encourage help-seeking: 71% of respondents overall, and 97% of Black respondents, rated this as “very important” (Appendix Table 9). Beyond emergency response, 988 also represents a step toward a broader “community care” system that trains and empowers lay caregivers to provide support alongside licensed medical and mental health professionals (Draper & Reinhart, 2025). By employing local community members on hotlines, mobile crisis teams, and in public mental health centers, for example, community care models shift emphasis away from high-cost, reactive medical interventions and reliance on external actors toward building local capacity for care. This approach restores to communities, particularly those with the greatest unmet social, medical, and economic needs, the resources and trust required to build systems of care from within.

Given longstanding disinvestment and harmful interactions with law enforcement in communities of color (GenForward & Movement for Black Lives, 2023), it is encouraging that Black adults in our study demonstrated greater awareness (35%) and willingness to use 988 than most other racial and ethnic subgroups, with more than 80% of Black respondents indicating they would be somewhat or very likely to contact 988 if they or someone they know were experiencing a crisis. Amid rising suicide rates among Black populations in recent years (D. M. Stone et al., 2023), it is critical that growing awareness and trust in 988 be backed by meaningful action and supportive services, including transparency around when and how contacting 988 may lead to encounters with law enforcement and public investment in community-based social infrastructure.

Another notable finding from this study is that, despite higher levels of awareness, younger individuals may be less inclined than older adults to contact 988. This pattern warrants further investigation, including an exploration of age-specific preferences for 988’s call, text, and chat modalities. Publicly available data on 988 use by modality and age are limited nationally and in California. Reporting from a few states suggests that chat and text are more popular among youth and young adults; however, age is unknown for a substantial proportion of contacts (30% or more) (Helpline Center, n.d.; Louisiana Department of Health, n.d.). Given these data limitations, one possible explanation for younger adults’ lower reported likelihood of contacting 988 is that greater familiarity with 988 may also be accompanied by increased awareness of potential risks or unintended consequences. In a separate survey of 5,451 US teens and adults, respondents aged 13–34 more often expressed concern about contacting 988 due to the possibility of law enforcement involvement, being taken to a hospital, or incurring costs than adults aged 49 and older (Ad Council Research Institute, 2023). Even more prevalent among this younger age group, however, were concerns about “opening up to a stranger” and the possibility of not feeling understood by the crisis counselor, highlighting the importance of building trust, cultural competence, and genuine human connection into crisis services (Ad Council Research Institute, 2023).

Reflecting this need, nearly 90% of survey respondents in the current study, and 95% of LGBT+ respondents, said that connecting to a crisis counselor with a similar background was a somewhat or very important consideration in their decision to contact 988 (Appendix Table 11). Grounded in the understanding that shared lived experiences, identities, and values can promote trust and engagement, the launch of 988 was accompanied by federal funding for specialized services serving high-risk groups, including veterans and LGBT+ youth and young adults, accessible through 988. Rather than being routed to a local crisis center, callers who pressed 1 or 3 after dialing 988 were connected to the Veterans Crisis Line or to a dedicated subnetwork providing LGBT+ counseling services, respectively, where they could speak with counselors specially trained to deliver compassionate, affirming care tailored to their needs and/or identities and often informed by similar life experiences. These specialized services and partnerships may help explain the relatively higher – though still limited – awareness of 988 among LGBT + and veteran respondents in our study, as well as the greater reported likelihood of use among LGBT+ individuals, despite concerns documented elsewhere (Ad Council Research Institute, 2023; Leigh et al., 2024). However, in the time since the current survey was conducted, the Trump Administration has discontinued the specialized services for LGBT+ individuals (effective July 17, 2025), a move that threatens to undermine the sense of safety and trust that LGBT+ help-seekers may have placed in the 988 system over the past several years (Chatterjee & Simmons-Duffin, 2025).

The finding that gun owners reported being just as likely as non-owners to utilize 988 is noteworthy, as it underscores the broad appeal of suicide prevention strategies that emphasize autonomy, privacy, and community- or peer-based support. Research indicates that individuals who die by firearm suicide are less likely than those using other methods to have previously sought or been receiving mental health treatment at the time of their death (Bond et al., 2022). Survey studies also show that gun owners are generally less supportive than non-owners of universal clinical interventions, such as firearm safety counseling (Pallin et al., 2019), and many firearm policies, such as prohibitions, licensure requirements, bans on assault weapons and high-capacity magazines, and mandatory safe storage laws (E. M. Stone et al., 2022), designed to prevent injury and violence. Yet in specific risk-based scenarios, such as a suicidal crisis, gun owners are equally or even more likely than non-owners to endorse interventions to prevent harm, particularly those that can be initiated by the person at risk or their family (Kravitz-Wirtz et al., 2021; Pallin et al., 2019). In this context, 988 may represent a promising strategy for reaching gun owners in need of mental health support. Given the lethality of firearms in suicide attempts, 988 crisis counselors should be prepared to discuss options for reducing access to firearms (and other lethal means), using a harm reduction approach that prioritizes collaboration and consent over coercion.

Regional variation in awareness of and willingness to use 988 highlights opportunities for enhanced public health efforts in areas most impacted by suicide. In more rural, northern regions of California where suicide rates are highest, such as the North Coast and Superior California, 988 awareness remains low; yet residents in these regions were among the most open to using 988 (Fig. 2 and Appendix Table 3). Raising awareness in these regions could improve access to crisis care and reduce suicides, especially if education and outreach strategies are tailored to the distinct needs and values of rural communities and delivered by messengers with locally derived influence and credibility.

Among the issues affecting whether someone might contact 988, respondents identified free and fast support as the most important factors influencing their decision. This finding aligns with national survey data (Ad Council Research Institute, 2023; National Alliance on Mental Illness (NAMI), 2024) and reinforces the critical role of incorporating low-barrier, 24/7 crisis lines within the conventional mental healthcare system, which can present significant structural, social, and financial impediments to care for many. While forced hospitalization and caller anonymity were rated as less important considerations overall, these factors may hold particular significance in some communities due to cultural norms, stigma surrounding mental health, and distrust of institutions (Misra et al., 2021). For example, 80% of Asian respondents – compared with 26% of White respondents – said it was “very important” to them that the crisis counselor would not know the identity of the person in crisis (Appendix Table 10). In addition, it is important to note that the survey did not distinguish between contacting 988 for oneself or someone else. It is possible that individuals may view privacy risks or the potential for involuntary intervention as more acceptable when seeking care for a loved one than when seeking care for themselves.

### Limitations

This study has several limitations that should be considered when interpreting the findings. First, as with most self-reported survey research, responses may have been influenced by social desirability bias, potentially leading to overestimates of reported awareness of 988 and likelihood of use. Further, while respondents reported a high likelihood of using 988, it is unclear to what extent these intentions translate into actual help-seeking behavior. Nevertheless, behavioral intentions are an important precursor to behavior and may still offer meaningful insights into public perceptions and openness to the service.

Second, given the low prevalence of 988 use in the general population (Purtle & Lindsey, 2025), the current survey did not assess respondents’ actual use of 988 or their current suicidal ideation or psychological distress, limiting our ability to understand how perceptions may differ between those who have and have not used 988 or who may need services most acutely. To assess program effectiveness, future research should expand on the limited body of work that centers the voices and experiences of individuals with direct or lived experience, particularly within disproportionately impacted communities (Britton et al., 2025; Gould et al., 2025).

Third, although survey weights adjusted for differential probability of selection and nonresponse, residual bias may remain if respondents differ systematically from nonrespondents in ways not fully captured by weighting adjustments. While item-level refusal was minimal (< 1%), analytic sample sizes were smaller for certain variables due to survey design features. Approximately 10% of respondents were missing data for LGBT + and veteran status because these measures were drawn from supplemental KnowledgePanel surveys administered on a rolling basis and were thus not available for all respondents. In addition, only the first 250 respondents were asked about perceived facilitators and barriers to 988 use; these items were later dropped due to survey length constraints. Larger samples would support more precise and robust analyses.

Fourth, this study was conducted in California, and while the state’s size and diversity make it a valuable context for examining public awareness and perceptions, the findings may not be generalizable to other states with different populations, policies, or mental healthcare infrastructures. However, because 988 systems are primarily administered at the state level, state-specific data remain critical to informing implementation efforts.

Finally, the survey focused exclusively on adults. Given the elevated and increasing prevalence of suicidal thoughts and behaviors among adolescents (Substance Abuse and Mental Health Services Administration, 2024), it is essential that future research explore awareness, attitudes, and experiences with 988 among youth populations.

## Conclusion

Since the launch of 988, increasing call volume has demonstrated both the substantial demand for and the potential lifesaving impacts of crisis prevention and intervention services, particularly those that are timely, confidential, free, and easy to access. However, realizing this potential requires additional efforts to increase public awareness and address barriers to accessing care, especially among those at greatest risk. As awareness and use of 988 continue to grow, sustained investments are needed to strengthen the broader crisis response system; this includes reliable funding for 988, comprehensive training and support for crisis counselors, and the development of a robust continuum of care that not only ensures emergency response aligns with caller needs but also extends beyond the moment of crisis and connects people to ongoing mental health services and community-based resources. Importantly, because mental health crises are often rooted in systemic inequities, suicide prevention must also involve structural change, including supporting people’s basic social needs such as housing, food, and healthcare, and dismantling the systems that produce and perpetuate inequality and harm.

## Supplementary Information

Below is the link to the electronic supplementary material.

Supplementary Material 1

## Data Availability

Data for this study will be made available when CSaWS 2024 analyses are complete.
