# Supplementary material for: Public Awareness and Perceptions of the 988 Suicide & Crisis Lifeline Among California Adults
Source: Community Ment Health J. 2026 Apr 28;62(7):1240–51. doi: 10.1007/s10597-026-01615-8 (PMC13586021; doi:10.1007/s10597-026-01615-8)
Supplement: Supplementary file 1 [file 10597_2026_1615_MOESM1_ESM.docx]

**Appendix for “Public awareness and perceptions of the 988 Suicide & Crisis Lifeline among California adults”**

Table of Contents

[Questions about 988 from the California Safety and Wellbeing Survey, 2024	3](#_Toc222315508)

[Methods for calculating average annual suicide rates in California regions	5](#_Toc222315509)

[Appendix Table 1. Sociodemographic characteristics of subsample and overall sample, 2024 California Safety and Wellbeing Survey	6](#_Toc222315510)

[Appendix Table 2. Likelihood of contacting 988, overall and by sociodemographic characteristics (n=3531)	8](#_Toc222315511)

[Appendix Table 3. Suicide rates and awareness and likelihood of using 988, by California region	10](#_Toc222315512)

[Appendix Table 4. Importance of factors in decision to contact 988 (n=250)	12](#_Toc222315513)

[Appendix Table 5. Importance of knowing that the person in crisis would be able to speak with a crisis counselor immediately, by sociodemographic characteristics (n=250)	13](#_Toc222315514)

[Appendix Table 6. Importance of knowing that the person in crisis would not receive a bill for contacting 988, by sociodemographic characteristics (n=250)	15](#_Toc222315515)

[Appendix Table 7. Importance of knowing that the person in crisis would not be forced to go to the hospital, by sociodemographic characteristics (n=250)	17](#_Toc222315516)

[Appendix Table 8. Importance of knowing that the person in crisis would not be taken to jail, by sociodemographic characteristics (n=250)	19](#_Toc222315517)

[Appendix Table 9. Importance of knowing that mental health professionals would be sent to the scene in cases of imminent risk, by sociodemographic characteristics (n=250)	21](#_Toc222315518)

[Appendix Table 10. Importance of knowing that the identity of the person in crisis would not be known by the person answering the call, by sociodemographic characteristics (n=250)	23](#_Toc222315519)

[Appendix Table 11. Importance of knowing that the person in crisis would be connected to a crisis counselor with a similar background, by sociodemographic characteristics (n=250)	25](#_Toc222315520)

[Appendix Table 12. Importance of knowing that the person in crisis would be connected to a crisis counselor who is from their state or local area, by sociodemographic characteristics (n=250)	27](#_Toc222315521)

[Appendix Table 13. Importance of knowing that police would be sent to the scene in cases of imminent risk, by sociodemographic characteristics (n=250)	29](#_Toc222315522)

[Appendix Table 14. Importance of knowing that the person in crisis would be able to receive support in a language other than English or Spanish, by sociodemographic characteristics (n=250)	31](#_Toc222315523)

# **Questions about 988 from the California Safety and Wellbeing Survey, 2024**

Q1. You likely have heard of some 3-digit telephone lines, such as 911 and 311. Have you also heard of the 3-digit telephone line, 988?

1. Yes

2. No

*988 is the 3-digit Suicide & Crisis Lifeline that people can contact if they or someone they know is experiencing a suicidal, substance use, or mental health crisis or is in emotional distress. 988 offers free and confidential 24/7 call, text, and chat support from trained crisis counselors. Crisis counselors may contact emergency responders in cases of imminent risk, only when a less invasive plan for safety cannot be established.*

Q2. How unlikely or likely would you be to contact 988 if you or someone you know was experiencing a suicidal, substance use, or mental health crisis, or was in emotional distress?

1. Very unlikely
2. Somewhat unlikely

3. Somewhat likely

4. Very likely

Q3. When considering whether to contact 988, how important are the following things to you?

*Statements in row [order randomized]:*

1. Knowing that the person in crisis would be able to speak with a crisis counselor immediately

2. Knowing that the person in crisis would not receive a bill for contacting 988

3. Knowing that the person in crisis would not be forced to go to the hospital

4. Knowing that the person in crisis would not be taken to jail

5. Knowing that mental health professionals would be sent to the scene in cases of imminent risk

6. Knowing that the identity of the person in crisis would not be known by the person answering the call

7. Knowing that the person in crisis would be connected to a crisis counselor with a similar background

8. Knowing that the person in crisis would be connected to a crisis counselor who is from their state or local area

9. Knowing that police would be sent to the scene in cases of imminent risk

10. Knowing that the person in crisis would be able to receive support in a language other than English or Spanish

*Answers in column:*

1. Very important

2. Somewhat important

3. Not at all important

# **Methods for calculating average annual suicide rates in California regions**

We computed the average annual suicide rates in each of California’s 10 Census regions using the following approach. We retrieved annual county-level counts of deaths from suicide (defined by ICD-10 codes: *U03, X60-X74, X75-X84, Y87.0) between January 1, 2018 and December 31, 2022 using the publicly available [California Department of Public Health – Vital Records Data (Cal-ViDa) query tool](https://cal-vida.cdph.ca.gov/VSQWeb), and county populations from the publicly available 2018-2022 American Community Survey 5-year estimates.

In accordance with the [Data De-Identification Guidelines](https://www.cdph.ca.gov/Programs/CHSI/CDPH%20Document%20Library/Help_and_FAQ_10.02.2020.pdf) established by the California Health and Human Services Agency, cell sizes between 1 and 10 are reported as “<11” in Cal-ViDa to protect individuals’ identities. To produce suicide estimates for these suppressed small numbers, we used a single imputation technique adapted from Erdman et al. (2021) that used the most recent non-missing data in years prior to and/or after a suppressed cell to inform replacement. For a county-year with a suppressed suicide count, if the county count in both years directly prior and after was 11+, the missing count was set as 10; if only one of the bordering years had a count of 11+ and the other was suppressed, the missing count was set as half of the available year, not to exceed 10; if both years directly prior and after were suppressed, the counts from two years before and after were examined and the previous steps applied; and for counties that had suppressed cells for all years, all years were conservatively assigned a suicide count of 1. The summed county-level totals following imputation differed by less than 1% from reported statewide suicide counts in all years.

To generate regional suicide rates (per 100,000 people), we divided the sum of the average annual number of suicides during 2018-2022 in each county within each region by the sum of the county populations within each region and multiplied by 100,000.

# Appendix Table 1. Sociodemographic characteristics of subsample and overall sample, 2024 California Safety and Wellbeing Survey

|  | **Subsample (n=250)**  Weighted % (95% CI) [unweighted n] | **Overall sample (n=3531)**  Weighted % (95% CI) [unweighted n] |
| --- | --- | --- |
| Sex |  |  |
| Female | 52.3 (42.6-61.8) [126] | 51.8 (49.3-54.3) [1943] |
| Male | 47.7 (38.2-57.4) [124] | 48.2 (45.7-50.7) [1943] |
| Age |  |  |
| 18-29 | 16.1 (8.7-28.0) [13] | 14.6 (12.6-17.0) [213] |
| 30-44 | 27.0 (18.9-37.1) [45] | 31.0 (28.6-33.6) [673] |
| 45-59 | 27.8 (20.1-37.0) [62] | 25.4 (23.4-27.6) [891] |
| 60+ | 29.1 (22.5-36.6) [130] | 28.9 (27.0-30.9) [1754] |
| Race/ethnicity |  |  |
| Asian | 15.3 (9.0-24.7) [21] | 15.4 (13.5-17.5) [314] |
| Black | 4.7 (2.1-10.0) [16] | 5.2 (4.2-6.5) [215] |
| Latine | 32.9 (23.8-43.42) [61] | 35.9 (33.5-38.4) [1118] |
| White | 41.4 (32.7-50.6) [144] | 39.7 (37.3-42.1) [1783] |
| Other/multi-race | 5.9 (2.3-14.3) [8] | 3.8 (2.9-5.0) [101] |
| Household income |  |  |
| Less than $50,000 | 18.0 (11.9-26.1) [66] | 21.8 (19.9-23.7) [1161] |
| $50,000 to $99,999 | 19.2 (13.0-27.3) [75] | 25.9 (23.7-28.1) [1013] |
| $100,000 to $149,999 | 19.8 (13.5-28.1) [43] | 19.6 (17.6-21.7) [587] |
| $150,000 or more | 43.1 (33.6-53.2) [66] | 32.8 (30.4-35.3) [770] |
| Region |  |  |
| Central Coast | 7.0 (4.2-11.5) [23] | 6.0 (5.0-7.2) [242] |
| Inland Empire | 12.0 (6.8-20.1) [29] | 10.8 (9.3-12.4) [419] |
| Los Angeles County | 23.6 (16.2-33.2) [47] | 26.1 (23.9-28.5) [826] |
| North Coast | 4.3 (1.4-12.2) [7] | 2.7 (2.1-3.5) [109] |
| Northern San Joaquin Valley | 3.8 (2.0-7.0) [14] | 4.6 (3.7-5.7) [175] |
| Orange County | 8.3 (4.8-13.8) [20] | 8.2 (6.9-9.7) [237] |
| San Diego - Imperial | 9.0 (5.3-14.9) [32] | 8.6 (7.3-10.0) [326] |
| San Francisco Bay Area | 18.6 (11.4-29.0) [39] | 18.7 (16.8-20.8) [606] |
| Southern San Joaquin Valley | 5.5 (2.5-11.7) [17] | 5.9 (4.8-7.0) [264] |
| Superior California | 8.0 (4.0-15.2) [22] | 8.5 (7.2-10.0) [327] |
| Gun ownership status |  |  |
| Gun owner | 15.0 (9.8-22.3) [46] | 15.4 (13.8-17.2) [618] |
| Non-owner who lives with owner | 10.1 (5.4-18.0) [23] | 11.3 (9.6-13.1) [333] |
| No guns in home | 72.1 (63.0-79.7) [174] | 67.0 (64.5-69.3) [2399] |
| Don’t know | 2.8 (1.2-7.3) [7] | 5.5 (4.3-6.9) [162] |
| Refused | 0 | 0.9 (0.5-1.6) [19] |
| LGBT+ |  |  |
| Yes^a^ | 7.5 (3.3-16.0) [20] | 11.2 (9.6-13.0) [329] |
| No (Heterosexual, cisgender) | 79.9 (70.0-87.1) [201] | 76.2 (73.8-78.4) [2797] |
| Not asked | 9.2 (5.0-16.2) [27] | 10.8 (9.3-12.5) [365] |
| Refused | 3.5 (0.8-13.9) [2] | 1.8 (1.2-2.8) [40] |
| Veteran |  |  |
| Yes | 6.3 (3.9-9.8) [28] | 4.9 (4.1-5.8) [270] |
| No | 84.4 (77.2-89.7) [197] | 84.3 (82.4-86.0) [2897] |
| Not asked | 8.3 (4.3-15.4) [24] | 10.3 (8.8-12.0) [349] |
| Refused | 1.0 (0.1-6.9) [1] | 0.5 (0.3-1.0) [15] |

Notes: ^a^LGBT+ includes respondents who identified as lesbian, gay, bisexual, transgender, non-binary, or some “other” sexual orientation or gender identity.

# Appendix Table 2. Likelihood of contacting 988, overall and by sociodemographic characteristics (n=3531)

|  | **Very likely**  % (95% CI) [n] | **Somewhat likely**  % (95% CI) [n] | **Somewhat unlikely**  % (95% CI) [n] | **Very unlikely**  % (95% CI) [n] |
| --- | --- | --- | --- | --- |
| Total | 30.2 (28.0-32.5) [1202] | 41.3 (38.8-43.7) [1454] | 15.8 (13.9-17.8) [442] | 12.2 (10.6-13.9) [415] |
| Sex |  |  |  |  |
| Female | 36.0 (32.8-39.3) [748] | 37.0 (33.8-40.2) [742] | 15.0 (12.5-17.8) [226] | 11.3 (9.3-13.7) [219] |
| Male | 24.0 (21.1-27.3) [454] | 45.8 (42.1-49.6) [712] | 16.6 (13.8-19.8) [216] | 13.0 (10.7-15.7) [196] |
| Age |  |  |  |  |
| 18-29 | 26.9 (20.0-35.1) [55] | 38.8 (31.1-47.2) [89] | 20.0 (14.0-27.7) [37] | 14.0 (9.3-20.5) [30] |
| 30-44 | 21.6 (17.8-25.9) [172] | 43.9 (38.9-49.1) [277] | 21.7 (17.8-26.3) [137] | 12.0 (9.0-15.8) [82] |
| 45-59 | 34.8 (30.4-39.4) [302] | 39.7 (35.3-44.2) [366] | 12.0 (9.0-15.7) [97] | 12.8 (10.0-16.1) [123] |
| 60+ | 37.1 (33.9-40.5) [673] | 40.9 (37.7-44.3) [722] | 10.6 (8.5-13.0) [171] | 10.9 (8.8-13.4) [180] |
| Race/ethnicity |  |  |  |  |
| Asian | 21.4 (16.4-27.5) [83] | 48.1 (40.8-55.4) [144] | 20.6 (15.1-27.5) [50] | 9.9 (6.3-15.3) [37] |
| Black | 42.8 (32.1-54.1) [99] | 38.6 (28.2-50.1) [67] | 9.0 (4.4-17.6) [16] | 8.8 (5.5-13.8) [31] |
| Latine | 29.8 (25.9-33.9) [375] | 35.0 (31.0-39.3) [393] | 15.7 (12.8-19.3) [158] | 18.9 (15.7-22.6) [187] |
| White | 32.8 (29.6-36.2) [616] | 44.4 (41.0-47.9) [801] | 13.7 (11.3-16.6) [204] | 8.0 (6.3-10.2) [151] |
| Other/multi-race | 25.6 (15.4-39.5) [29] | 42.9 (29.9-56.9) [49] | 27.0 (14.7-44.1) [14] | 4.5 (1.9-10.2) [9] |
| Household income |  |  |  |  |
| Less than $50,000 | 29.9 (25.8-34.3) [413] | 33.6 (29.3-38.1) [413] | 15.0 (11.8-18.9) [141] | 20.7 (16.8-25.2) [186] |
| $50,000 to $99,999 | 29.8 (25.5-34.4) [339] | 41.9 (37.2-46.8) [426] | 12.8 (10.0-16.3) [123] | 14.5 (11.2-18.5) [119] |
| $100,000 to $149,999 | 27.7 (23.1-32.8) [189] | 44.6 (39.0-50.4) [267] | 19.4 (14.7-25.1) [78] | 8.2 (5.6-12.0) [51] |
| $150,000 or more | 32.3 (28.1-36.9) [261] | 43.8 (39.2-48.5) [348] | 16.5 (13.0-20.7) [100] | 7.0 (5.1-9.5) [59] |
| Gun ownership status |  |  |  |  |
| Gun owner | 28.4 (23.5-33.9) [174] | 44.5 (38.8-50.4) [288] | 16.6 (12.0-22.4) [75] | 10.3 (7.5-14.0) [77] |
| Non-owner who lives with owner | 39.3 (31.4-47.7) [128] | 35.5 (28.3-43.5) [136] | 18.1 (12.3-25.9) [44] | 7.1 (4.1-11.9) [25] |
| No guns in home | 29.5 (26.9-32.2) [848] | 42.3 (39.3-45.4) [969] | 14.8 (12.6-17.2) [291] | 12.9 (11.0-15.1) [284] |
| LGBT+ |  |  |  |  |
| Yes^a^ | 33.4 (26.1-41.6) [106] | 43.9 (36.0- 52.0) [141] | 16.7 (11.8-23.2) [54] | 6.0 (3.7-9.8) [28] |
| No (Heterosexual, cisgender) | 29.6 (27.2-32.2) [958] | 41.6 (38.8-44.3) [1177] | 16.4 (14.2-18.9) [333] | 12.2 (10.5-14.2) [321] |
| Veteran |  |  |  |  |
| Yes | 30.6 (23.5-38.7) [94] | 39.1 (30.8-48.0) [111] | 18.4 (11.3-28.6) [32] | 11.7 (7.1-18.5) [30] |
| No | 29.8 (27.4-32.4) [981] | 41.9 (39.2-44.7) [1220] | 16.0 (13.9-18.3) [357] | 11.9 (10.3-13.8) [331] |

Notes: ^a^LGBT+ includes respondents who identified as lesbian, gay, bisexual, transgender, non-binary, or some “other” sexual orientation or gender identity.

Row percentages may not sum to 100% because refusals are not shown.

# Appendix Table 3. Suicide rates and awareness and likelihood of using 988, by California region

| **Region** | **Counties within region** | **Aware of 988,** Weighted % (95% CI)  [n] | **Somewhat or very likely to use 988,** Weighted %  (95% CI)  [n] | **Average annual suicide rate per 100,000**  **(2018-22)** | **Average annual suicide count (2018-22)** |
| --- | --- | --- | --- | --- | --- |
| Central Coast | Monterey, San Benito, San Luis Obispo, Santa Barbara, Santa Cruz, Ventura | 16.6  (11.4-23.6)  [50] | 72.9  (63.7-80.5)  [183] | 12.0 | 281 |
| Inland Empire | Riverside, San Bernardino | 28.2  (21.6-35.8)  [97] | 75.0  (67.8-81.1)  [313] | 11.6 | 533 |
| Los Angeles County | Los Angeles | 26.1  (21.8-31.0)  [209] | 65.5  (60.2-70.4)  [604] | 8.8 | 872 |
| North Coast | Del Norte, Humboldt, Lake, Mendocino, Napa, Sonoma, Trinity | 22.5  (13.8-34.6)  [27] | 77.8  (62.5-88.1)  [90] | 16.9 | 163 |
| Northern San Joaquin Valley | Alpine, Amador, Calaveras, Madera, Mariposa, Merced, Mono, San Joaquin, Stanislaus, Tuolumne | 27.6  (18.6-38.9)  [44] | 64.5  (53.2-74.4)  [126] | 12.0 | 233 |
| Orange County | Orange | 24.6  (17.2-33.8)  [49] | 72.1  (63.5-79.3)  [179] | 10.7 | 340 |
| San Diego - Imperial | Imperial, San Diego | 29.0  (22.0-37.2)  [68] | 72.4  (64.5-79.1)  [245] | 11.9 | 413 |
| San Francisco Bay Area | Alameda, Contra Costa, Marin, San Francisco, San Mateo, Santa Clara, Solano, Solano | 28.6  (23.5-34.4)  [167] | 76.3  (70.5-81.2)  [476] | 9.7 | 683 |
| Southern San Joaquin Valley | Fresno, Inyo, Kern, Kings, Tulare | 30.3  (22.8-39.1)  [83] | 68.4  (58.5-76.8)  [187] | 10.8 | 277 |
| Superior California | Butte, Colusa, El Dorado, Glenn, Lassen, Modoc, Nevada, Placer, Plumas, Sacramento, Shasta, Sierra, Siskiyou, Sutter, Tehama, Yolo, Yuba, Del Norte | 25.7  (18.8-34.0)  [88] | 76.0  (67.7-82.8)  [253] | 14.9 | 491 |

# Appendix Table 4. Importance of factors in decision to contact 988 (n=250)

|  | **Very important**  % (95% CI) [n] | **Somewhat important**  % (95% CI) [n] | **Not at all important**  % (95% CI) [n] |
| --- | --- | --- | --- |
| Knowing that the person in crisis would be able to speak with a crisis counselor immediately | 79.0 (69.6-86.1) [206] | 19.7 (12.9-29.0) [42] | 0 |
| Knowing that the person in crisis would not receive a bill for contacting 988 | 72.3 (63.1-79.9) [182] | 21.7 (14.8-30.9) [47] | 4.7 (2.6-8.3) [19] |
| Knowing that mental health professionals would be sent to the scene in cases of imminent risk | 70.7 (60.9-78.9) [181] | 26.9 (19.0-36.8) [63] | 0.9 (0.2-3.7) [2] |
| Knowing that the person in crisis would not be taken to jail | 61.8 (52.1-70.7) [148] | 30.7 (22.4-40.5) [77] | 6.2 (3.5-10.8) [23] |
| Knowing that the person in crisis would be able to receive support in a language other than English or Spanish | 59.5 (50.0-68.3) [133] | 26.5 (19.2-35.3) [82] | 12.4 (7.7-19.3) [32] |
| Knowing that the person in crisis would be connected to a crisis counselor who is from their state or local area | 51.3 (41.7-60.9) [137] | 35.8 (26.9-45.8) [82] | 11.4 (6.8-18.4) [28] |
| Knowing that police would be sent to the scene in cases of imminent risk | 50.8 (41.1-60.4) [139] | 34.4 (25.7-44.3) [90] | 13.0 (6.8-23.6) [16] |
| Knowing that the person in crisis would be connected to a crisis counselor with a similar background | 47.4 (37.9-57.1) [123] | 40.0 (30.8-50.0) [100] | 11.3 (6.7-18.6) [25] |
| Knowing that the identity of the person in crisis would not be known by the person answering the call | 39.4 (30.4-49.2) [94] | 38.0 (29.1-47.8) [96] | 21.3 (14.3-30.4) [57] |
| Knowing that the person in crisis would not be forced to go to the hospital | 37.5 (28.4-47.5) [83] | 42.9 (33.7-52.5) [113] | 18.4 (11.9-27.4) [52] |

Notes: Row percentages may not sum to 100% because refusals are not shown.

# Appendix Table 5. Importance of knowing that the person in crisis would be able to speak with a crisis counselor immediately, by sociodemographic characteristics (n=250)

|  | **Very important**  % (95% CI) [n] | **Somewhat important**  % (95% CI) [n] | **Not at all important**  % (95% CI) [n] |
| --- | --- | --- | --- |
| Sex |  |  |  |
| Female | 80.1 (66.0-89.3) [108] | 17.9 (9.2-32.0) [17] | 0 |
| Male | 77.8 (64.2-87.3) [98] | 21.7 (12.3-35.3) [25] | 0 |
| Age |  |  |  |
| 18-29 | 75.7 (38.8-93.8) [11] | 24.3 (6.2-61.2) [2] | 0 |
| 30-44 | 74.0 (52.3-88.1) [34] | 26.0 (11.9-47.7) [11] | 0 |
| 45-59 | 82.5 (66.4-91.8) [51] | 17.5 (8.2-33.6) [11] | 0 |
| 60+ | 82.1 (70.9-89.7) [110] | 13.5 (7.7-22.5) [18] | 0 |
| Race/ethnicity |  |  |  |
| Asian | 60.9 (31.3-84.1) [17] | 39.1 (15.9-68.7) [4] | 0 |
| Black | 100 [16] | 0 | 0 |
| Latine | 80.5 (62.6-91.0) [47] | 19.5 (9.0-37.4) [14] | 0 |
| White | 79.2 (67.5-87.5) [118] | 17.7 (10.3-28.9) [24] | 0 |
| Other/multi-race | 100 [8] | 0 | 0 |
| Household income |  |  |  |
| Less than $50,000 | 68.5 (44.5-85.5) [52] | 31.5 (14.5-55.5) [14] | 0 |
| $50,000 to $99,999 | 82.9 (65.6-92.5) [63] | 17.1 (7.5-34.4) [12] | 0 |
| $100,000 to $149,999 | 82.8 (56.0-94.8) [37] | 16.0 (4.4-43.8) [5] | 0 |
| $150,000 or more | 79.9 (64.3-89.8) [54] | 17.7 (8.5-33.1) [11] | 0 |
| Gun ownership status |  |  |  |
| Gun owner | 82.7 (66.6-92.0) [36] | 15.7 (7.0-31.5) [9] | 0 |
| Non-owner who lives with owner | 93.2 (70.1-98.8) [21] | 6.8 (1.2-29.9) [2] | 0 |
| No guns in home | 78.4 (66.2-87.0) [146] | 20.2 (11.8-32.3) [27] | 0 |
| LGBT+ |  |  |  |
| Yes | 88.9 (63.1-97.4) [16] | 11.1 (2.6-36.9) [4] | 0 |
| No (Heterosexual, cisgender) | 77.0 (66.1-85.2) [165] | 22.7 (14.5-33.6) [35] | 0 |
| Veteran |  |  |  |
| Yes | 82.5 (61.6-93.2) [22] | 17.5 (6.8-38.4) [6] | 0 |
| No | 78.5 (67.8-86.4) [163] | 21.2 (13.3-31.9) [33] | 0 |

Notes: Row percentages may not sum to 100% because refusals are not shown.

# Appendix Table 6. Importance of knowing that the person in crisis would not receive a bill for contacting 988, by sociodemographic characteristics (n=250)

|  | **Very important**  % (95% CI) [n] | **Somewhat important**  % (95% CI) [n] | **Not at all important**  % (95% CI) [n] |
| --- | --- | --- | --- |
| Sex |  |  |  |
| Female | 66.0 (52.2-77.6) [93] | 27.0 (16.4-41.0) [24] | 5.0 (2.2-11.3) [8] |
| Male | 79.2 (67.3-87.5) [89] | 16.0 (8.6-28.0) [23] | 4.3 (1.9-9.5) [11] |
| Age |  |  |  |
| 18-29 | 75.7 (38.8-93.8) [11] | 24.3 (6.2-61.2) [2] | 0 |
| 30-44 | 84.3 (63.7-94.2) [35] | 14.5 (4.9-35.8) [7] | 1.2 (0.4-4.2) [3] |
| 45-59 | 73.9 (56.5-86.1) [47] | 23.7 (12.0-41.4) [12] | 2.3 (0.6-8.1) [3] |
| 60+ | 57.7 (45.9-68.7) [89] | 25.1 (16.3-36.7) [26] | 12.8 (6.6-23.3) [13] |
| Race/ethnicity |  |  |  |
| Asian | 61.0 (32.0-83.9) [15] | 35.6 (13.6-66.0) [5] | 3.4 (0.5-21.4) [1] |
| Black | 100 [16] | 0 | 0 |
| Latine | 78.8 (61.6-89.6) [41] | 18.6 (8.4-36.1) [14] | 2.7 (1.0-6.6) [6] |
| White | 66.2 (54.3-76.4) [103] | 22.7 (14.2-34.2) [27] | 8.0 (3.9-15.7) [12] |
| Other/multi-race | 86.1 (39.8-98.3) [7] | 13.9 (1.7-60.2) [1] | 0 |
| Household income |  |  |  |
| Less than $50,000 | 78.1 (54.4-91.4) [52] | 21.9 (8.6-45.6) [14] | 0 |
| $50,000 to $99,999 | 70.7 (53.1-83.7) [50] | 17.3 (7.8-34.2) [12] | 12.0 (5.6-24.0) [13] |
| $100,000 to $149,999 | 69.4 (46.8-85.3) [32] | 22.5 (8.5-47.8) [6] | 6.9 (2.1-19.9) [4] |
| $150,000 or more | 71.9 (56.4-83.5) [48] | 23.3 (12.8-38.6) [15] | 2.4 (0.5-9.9) [2] |
| Gun ownership status |  |  |  |
| Gun owner | 60.9 (40.3-78.2) [30] | 26.3 (12.7-46.7) [10] | 11.2 (4.0-27.3) [5] |
| Non-owner who lives with owner | 87.4 (66.4-96.1) [17] | 10.1 (2.7-31.4) [4] | 2.5 (0.4-13.5) [2] |
| No guns in home | 74.6 (63.0-83.5) [133] | 20.1 (11.9-31.9) [28] | 3.8 (1.8-8.1) [12] |
| LGBT+ |  |  |  |
| Yes | 88.7 (64.0-97.2) [15] | 9.3 (2.0-34.7) [3] | 2.0 (0.3-10.7) [15] |
| No (Heterosexual, cisgender) | 69.4 (58.9-78.2) [146] | 25.7 (17.3-36.4) [39] | 4.6 (2.3-8.7) [2] |
| Veteran |  |  |  |
| Yes | 83.3 (61.0-94.1) [22] | 7.7 (2.5-21.4) [4] | 9.0 (1.6-37.0) [2] |
| No | 71.1 (60.7-79.7) [142] | 24.8 (16.6-35.3) [39] | 3.8 (1.9-7.5) [15] |

Notes: Row percentages may not sum to 100% because refusals are not shown.

# Appendix Table 7. Importance of knowing that the person in crisis would not be forced to go to the hospital, by sociodemographic characteristics (n=250)

|  | **Very important**  % (95% CI) [n] | **Somewhat important**  % (95% CI) [n] | **Not at all important**  % (95% CI) [n] |
| --- | --- | --- | --- |
| Sex |  |  |  |
| Female | 37.4 (25.0-51.7) [43] | 41.2 (29.0-54.5) [59] | 19.4 (10.0-34.4) [23] |
| Male | 37.5 (25.1-51.7) [40] | 44.7 (31.7-58.5) [54] | 17.3 (10.1-28.0) [29] |
| Age |  |  |  |
| 18-29 | 22.3 (6.6-53.9) [6] | 49.7 (20.4-79.2) [5] | 28.1 (7.4-65.6) [2] |
| 30-44 | 51.1 (31.7-70.2) [19] | 38.8 (22.3-58.4) [21] | 10.0 (3.2-27.4) [5] |
| 45-59 | 42.4 (26.1-60.6) [24] | 38.3 (23.6-55.5) [25] | 19.3 (9.1-36.5) [13] |
| 60+ | 28.4 (19.5-39.4) [34] | 47.2 (36.2-58.4) [62] | 20.0 (13.1-29.4) [32] |
| Race/ethnicity |  |  |  |
| Asian | 25.6 (10.5-50.0) [9] | 52.1 (26.2-76.9) [10] | 22.4 (5.8-57.4) [2] |
| Black | 61.6 (28.0-86.9) [8] | 24.3 (7.2-56.9) [5] | 14.1 (3.6-41.8) [3] |
| Latine | 43.1 (25.6-62.5) [24] | 41.0 (23.8-60.8) [25] | 15.9 (5.8-36.7) [12] |
| White | 31.1 (20.5-44.2) [39] | 43.7 (32.9-55.2) [68] | 22.1 (14.0-33.0) [35] |
| Other/multi-race | 62.4 (22.5-90.4) [3] | 37.6 (9.6-77.5) [5] | 0 |
| Household income |  |  |  |
| Less than $50,000 | 59.0 (38.6-76.7) [30] | 33.0 (17.3-53.8) [29] | 8.0 (3.0-19.7) [7] |
| $50,000 to $99,999 | 24.1 (13.3-39.5) [23] | 59.1 (41.1-74.9) [28] | 16.8 (9.4-28.4) [24] |
| $100,000 to $149,999 | 35.1 (19.9-54.3) [14] | 40.0 (22.5-60.4) [18] | 23.7 (9.6-47.5) [10] |
| $150,000 or more | 35.5 (20.9-53.4) [16] | 41.0 (27.1-56.6) [38] | 21.1 (10.3-38.3) [11] |
| Gun ownership status |  |  |  |
| Gun owner | 32.4 (13.4-59.8) [8] | 42.5 (24.6-62.7) [25] | 23.5 (11.2-42.8) [12] |
| Non-owner who lives with owner | 63.8 (32.4-86.6) [12] | 30.0 (9.4-63.8) [7] | 6.2 (1.7-19.8) [4] |
| No guns in home | 36.3 (25.9-48.1) [63] | 42.7 (31.7-54.4) [75] | 19.6 (11.5-31.4) [35] |
| LGBT+ |  |  |  |
| Yes | 8.2 (2.3-25.5) [6] | 42.4 (12.4-79.3) [6] | 49.4 (15.6-83.7) [8] |
| No (Heterosexual, cisgender) | 36.1 (26.6-46.8) [62] | 47.0 (36.8-57.5) [101] | 16.6 (10.2-26.0) [37] |
| Veteran |  |  |  |
| Yes | 14.0 (5.431.7) [5] | 62.6 (40.3-80.5) [17] | 23.4 (9.1-48.2) [6] |
| No | 37.0 (27.1-48.1) [65] | 43.8 (33.5-54.7) [91] | 18.9 (11.6-29.3) [40] |

Notes: Row percentages may not sum to 100% because refusals are not shown.

# Appendix Table 8. Importance of knowing that the person in crisis would not be taken to jail, by sociodemographic characteristics (n=250)

|  | **Very important**  % (95% CI) [n] | **Somewhat important**  % (95% CI) [n] | **Not at all important**  % (95% CI) [n] |
| --- | --- | --- | --- |
| Sex |  |  |  |
| Female | 65.1 (51.5-76.6) [79] | 28.0 (17.4-41.7) [36] | 5.0 (2.4-9.9) [10] |
| Male | 58.3 (44.3-71.0) [69] | 33.6 (21.7-48.1) [41] | 7.6 (3.3-16.7) [13] |
| Age |  |  |  |
| 18-29 | 58.5 (25.8-85.1) [9] | 40.8 (14.4-73.8) [3] | 0.8 (0.1-5.8) [1] |
| 30-44 | 72.3 (52.4-86.1) [28] | 22.1 (10.3-41.3) [14] | 5.6 (1.0-25.7) [3] |
| 45-59 | 61.5 (43.9-76.6) [37] | 30.8 (17.0-49.2) [17] | 7.7 (3.4-16.6) [8] |
| 60+ | 54.2 (42.9-65.1) [74] | 33.0 (23.3-44.2) [43] | 8.5 (4.2-16.2) [11] |
| Race/ethnicity |  |  |  |
| Asian | 40.5 (18.7-66.8) [12] | 47.5 (22.4-73.8) [7] | 12.0 (2.6-41.0) [2] |
| Black | 95.8 (73.1-99.5) [15] | 0 | 4.2 (0.5-26.9) [1] |
| Latine | 64.2 (44.7-80.0) [32] | 33.5 (18.2-53.3) [24] | 2.3 (0.8-6.3) [5] |
| White | 60.1 (48.5-70.7) [83] | 29.9 (20.5-41.3) [45] | 6.9 (3.8-12.5) [14] |
| Other/multi-race | 89.0 (51.2-98.4) [6] | 1.3 (0.1-10.3) [1] | 9.7 (1.2-49.8) [1] |
| Household income |  |  |  |
| Less than $50,000 | 72.4 (50.7-87.1) [42] | 25.6 (11.5-47.8) [22] | 1.9 (0.4-8.2) [2] |
| $50,000 to $99,999 | 44.5 (26.8-63.8) [38] | 48.5 (29.5-67.9) [27] | 7.0 (3.2-14.3) [10] |
| $100,000 to $149,999 | 57.5 (37.2-75.5) [25] | 30.1 (14.2-52.7) [11] | 11.2 (4.6-25.2) [6] |
| $150,000 or more | 67.1 (51.0-80.0) [43] | 25.1 (14.0-41.0) [17] | 5.4 (1.6-16.5) [5] |
| Gun ownership status |  |  |  |
| Gun owner | 53.5 (33.0-72.9) [23] | 32.4 (17.1-52.8) [14] | 12.4 (5.4-26.0) [8] |
| Non-owner who lives with owner | 85.7 (64.1-95.3) [17] | 11.7 (3.4-32.8) [5] | 2.7 (0.3-17.8) [1] |
| No guns in home | 60.7 (48.7-71.6) [105] | 32.2 (21.9-44.5) [54] | 5.7 (2.6-12.0) [14] |
| LGBT+ |  |  |  |
| Yes | 80.4 (52.1-93.9) [13] | 13.6 (3.5-40.6) [4] | 6.0 (1.4-21.9) [3] |
| No (Heterosexual, cisgender) | 57.6 (46.9-67.7) [118] | 35.5 (25.8-46.6) [65] | 6.6 (3.4-12.3) [17] |
| Veteran |  |  |  |
| Yes | 47.1 (27.0-68.2) [13] | 31.3 (15.5-53.2) [10] | 21.6 (7.7-47.4) [5] |
| No | 61.9 (50.9-71.8) [121] | 32.7 (23.2-43.9) [60] | 5.1 (2.5-10.4) [15] |

Notes: Row percentages may not sum to 100% because refusals are not shown.

# Appendix Table 9. Importance of knowing that mental health professionals would be sent to the scene in cases of imminent risk, by sociodemographic characteristics (n=250)

|  | **Very important**  % (95% CI) [n] | **Somewhat important**  % (95% CI) [n] | **Not at all important**  % (95% CI) [n] |
| --- | --- | --- | --- |
| Sex |  |  |  |
| Female | 76.9 (63.2-86.5) [97] | 21.0 (11.8-34.5) [27] | 0 |
| Male | 64.0 (49.7-76.3) [84] | 33.5 (21.5-48.1) [36] | 1.8 (0.4-7.5) [2] |
| Age |  |  |  |
| 18-29 | 75.7 (38.8-93.8) [11] | 24.3 (6.2-61.2) [2] | 0 |
| 30-44 | 59.5 (38.2-77.7) [31] | 40.5 (22.3-61.8) [14] | 0 |
| 45-59 | 78.0 (62.1-88.5) [46] | 18.6 (9.0-34.4) [13] | 3.1 (0.7-12.6) [2] |
| 60+ | 71.5 (60.1-80.7) [93] | 23.7 (15.8-34.0) [34] | 0 |
| Race/ethnicity |  |  |  |
| Asian | 60.9 (31.4-84.1) [16] | 39.1 (15.9-68.6) [5] | 0 |
| Black | 97.1 (80.1-99.6) [15] | 2.9 (0.3-19.9) [1] | 0 |
| Latine | 78.9 (60.7-90.0) [45] | 21.1 (10.0-39.3) [16] | 0 |
| White | 70.5 (59.0-79.8) [101] | 25.3 (16.7-36.3) [38] | 0.7 (0.1-5.0) [1] |
| Other/multi-race | 31.7 (7.2-73.4) [4] | 58.6 (18.9-89.6) [3] | 9.7 (1.2-49.8) [1] |
| Household income |  |  |  |
| Less than $50,000 | 62.5 (40.1-80.5) [45] | 37.1 (19.1-59.6) [20] | 0 |
| $50,000 to $99,999 | 82.5 (65.8-92.1) [58] | 16.9 (7.5-33.8) [16] | 0 |
| $100,000 to $149,999 | 71.8 (49.1-87.0) [32] | 22.6 (8.7-47.1) [8] | 4.4 (1.0-17.2) [2] |
| $150,000 or more | 68.4 (51.7-81.5) [46] | 29.2 (16.6-46.0) [19] | 0 |
| Gun ownership status |  |  |  |
| Gun owner | 55.2 (33.0-75.4) [28] | 40.7 (20.7-64.3) [15] | 2.0 (0.3-13.2) [1] |
| Non-owner who lives with owner | 95.1 (82.6-98.7) [20] | 4.9 (1.3-17.4) [3] | 0 |
| No guns in home | 71.4 (59.6-80.9) [130] | 26.2 (17.0-38.0) [41] | 0.8 (0.1-5.4) [1] |
| LGBT+ |  |  |  |
| Yes | 76.0 (43.0-93.0) [14] | 24.0 (7.0-57.0) [6] | 0 |
| No (Heterosexual, cisgender) | 68.1 (57.0-77.6) [145] | 30.2 (20.9-41.5) [51] | 1.1 (0.3-4.5) [2] |
| Veteran |  |  |  |
| Yes | 62.2 (39.2-80.8) [19] | 28.7 (13.2-51.5) [8] | 9.1 (1.3-42.7) [1] |
| No | 70.0 (58.9-79.2) [143] | 29.1 (20.0-40.3) [50] | 0.4 (0.0-2.5) [1] |

Notes: Row percentages may not sum to 100% because refusals are not shown.

# Appendix Table 10. Importance of knowing that the identity of the person in crisis would not be known by the person answering the call, by sociodemographic characteristics (n=250)

|  | **Very important**  % (95% CI) [n] | **Somewhat important**  % (95% CI) [n] | **Not at all important**  % (95% CI) [n] |
| --- | --- | --- | --- |
| Sex |  |  |  |
| Female | 43.2 (30.3-57.0) [53] | 31.8 (20.9-45.2) [48] | 23.1 (12.9-37.7) [24] |
| Male | 35.3 (23.4-49.3) [41] | 44.8 (31.7-58.7) [48] | 19.3 (11.3-30.8) [33] |
| Age |  |  |  |
| 18-29 | 34.4 (12.2-66.5) [8] | 39.2 (13.5-72.7) [3] | 26.4 (6.8-63.9) [2] |
| 30-44 | 56.4 (36.4-74.5) [21] | 36.2 (19.5-57.1) [19] | 7.4 (2.7-18.8) [5] |
| 45-59 | 39.8 (24.3-57.7) [23] | 28.8 (16.3-45.7) [20] | 31.4 (17.0-50.5) [19] |
| 60+ | 25.9 (18.0-35.9) [42] | 47.8 (36.8-59.1) [54] | 21.7 (14.2-31.6) [31] |
| Race/ethnicity |  |  |  |
| Asian | 80.4 (59.1-92.1) [13] | 15.5 (5.7-36.0) [6] | 4.1 (0.7-20.0) [2] |
| Black | 65.1 (32.2-88.0) [7] | 7.3 (2.0-23.6) [4] | 27.6 (8.8-60.1) [5] |
| Latine | 27.4 (14.9-44.9) [22] | 51.7 (32.9-70.1) [26] | 20.9 (8.8-42.0) [13] |
| White | 26.3 (18.4-36.1) [48] | 42.1 (31.3-53.8) [58] | 28.4 (17.9-41.8) [35] |
| Other/multi-race | 71.9 (31.0-93.6) [4] | 15.2 (2.1-59.5) [2] | 12.9 (2.2-49.6) [2] |
| Household income |  |  |  |
| Less than $50,000 | 43.3 (24.3-64.4) [26] | 32.9 (16.9-54.1) [24] | 23.8 (10.1-46.7) [16] |
| $50,000 to $99,999 | 30.3 (17.5-47.1) [26] | 52.0 (33.1-70.4) [30] | 17.3 (9.1-30.5) [18] |
| $100,000 to $149,999 | 47.8 (28.9-67.3) [19] | 30.0 (14.7-51.5) [12] | 21.1 (10.2-38.6) [11] |
| $150,000 or more | 38.0 (23.6-54.9) [23] | 37.6 (24.1-53.4) [30] | 22.0 (10.6-40.1) [12] |
| Gun ownership status |  |  |  |
| Gun owner | 36.0 (16.6-61.3) [13] | 40.0 (22.7-60.3) [21] | 22.0 (10.0-41.6) [10] |
| Non-owner who lives with owner | 43.0 (18.4-71.6) [14] | 24.6 (6.3-61.3) [5] | 32.4 (9.3-69.0) [4] |
| No guns in home | 40.5 (29.7-52.4) [66] | 37.7 (27.0-49.7) [64] | 20.4 (12.6-31.3) [43] |
| LGBT+ |  |  |  |
| Yes | 10.5 (3.2-29.6) [7] | 44.1 (13.3-80.3) [7] | 45.4 (13.1-82.2) [6] |
| No (Heterosexual, cisgender) | 44.4 (34.2-55.1) [77] | 37.4 (28.0-47.9) [79] | 17.8 (11.6-26.3) [43] |
| Veteran |  |  |  |
| Yes | 21.4 (8.6-44.0) [5] | 39.5 (21.3-61.2) [12] | 39.1 (20.3-61.7) [11] |
| No | 41.4 (31.2-52.4) [79] | 39.7 (29.7-50.7) [76] | 18.5 (11.6-28.3) [40] |

Notes: Row percentages may not sum to 100% because refusals are not shown.

# Appendix Table 11. Importance of knowing that the person in crisis would be connected to a crisis counselor with a similar background, by sociodemographic characteristics (n=250)

|  | **Very important**  % (95% CI) [n] | **Somewhat important**  % (95% CI) [n] | **Not at all important**  % (95% CI) [n] |
| --- | --- | --- | --- |
| Sex |  |  |  |
| Female | 49.8 (36.4-63.3) [62] | 36.7 (24.7-50.7) [49] | 11.5 (5.3-23.0) [14] |
| Male | 44.8 (31.9-58.3) [61] | 43.6 (30.5-57.6) [51] | 11.2 (5.3-21.9) [11] |
| Age |  |  |  |
| 18-29 | 27.1 (8.1-61.2) [7] | 72.9 (38.8-91.9) [6] | 0 |
| 30-44 | 54.3 (34.6-72.8) [22] | 29.0 (15.6-47.6) [18] | 16.6 (5.8-39.0) [5] |
| 45-59 | 46.0 (29.5-63.5) [26] | 37.8 (22.4-56.0) [25] | 16.2 (7.8-30.7) [11] |
| 60+ | 53.5 (42.2-64.5) [68] | 34.1 (24.5-45.1) [51] | 8.0 (3.6-16.8) [9] |
| Race/ethnicity |  |  |  |
| Asian | 42.1 (19.7-68.4) [13] | 33.0 (12.3-63.3) [5] | 24.9 (7.6-57.1) [3] |
| Black | 78.8 (48.2-93.7) [11] | 3.6 (0.6-18.7) [2] | 17.6 (4.6-48.4) [3] |
| Latine | 41.5 (24.2-61.3) [25] | 50.2 (31.4-68.8) [32] | 8.3 (2.7-22.6) [4] |
| White | 46.7 (35.4-58.3) [70] | 40.6 (29.4-52.9) [57] | 9.6 (5.3-16.8) [15] |
| Other/multi-race | 74.3 (33.8-94.2) [4] | 25.7 (5.8-66.2) [4] | 0 |
| Household income |  |  |  |
| Less than $50,000 | 43.5 (25.1-64.0) [38] | 52.7 (32.5-72.1) [26] | 3.7 (0.8-15.2) [2] |
| $50,000 to $99,999 | 33.0 (19.5-50.0) [29] | 59.6 (41.4-75.5) [40] | 7.4 (2.5-20.2) [6] |
| $100,000 to $149,999 | 49.1 (30.2-68.3) [24] | 41.3 (22.7-62.9) [13] | 8.3 (2.7-23.0) [5] |
| $150,000 or more | 54.6 (38.7-69.7) [32] | 25.4 (14.5-40.5) [21] | 17.6 (8.6-32.7) [12] |
| Gun ownership status |  |  |  |
| Gun owner | 55.6 (35.3-74.2) [20] | 34.7 (19.3-54.2) [21] | 8.1 (2.5-23.0) [4] |
| Non-owner who lives with owner | 37.9 (15.7-66.7) [11] | 53.5 (25.0-79.9) [10] | 8.6 (1.9-31.7) [2] |
| No guns in home | 48.1 (36.7-59.8) [90] | 37.6 (26.8-49.8) [64] | 12.8 (6.9-22.5) [19] |
| LGBT+ |  |  |  |
| Yes | 56.1 (20.1-86.7) [11] | 38.4 (10.6-76.7) [7] | 5.4 (1.0-25.0) [2] |
| No (Heterosexual, cisgender) | 45.0 (34.9-55.4) [95] | 41.6 (31.5-52.4) [83] | 13.1 (7.5-21.9) [22] |
| Veteran |  |  |  |
| Yes | 39.8 (21.2-61.8) [12] | 48.0 (27.7-68.9) [14] | 12.3 (2.8-40.5) [2] |
| No | 48.0 (37.4-58.8) [97] | 39.2 (29.1-50.3) [76] | 12.5 (7.2-21.0) [23] |

Notes: Row percentages may not sum to 100% because refusals are not shown.

# Appendix Table 12. Importance of knowing that the person in crisis would be connected to a crisis counselor who is from their state or local area, by sociodemographic characteristics (n=250)

|  | **Very important**  % (95% CI) [n] | **Somewhat important**  % (95% CI) [n] | **Not at all important**  % (95% CI) [n] |
| --- | --- | --- | --- |
| Sex |  |  |  |
| Female | 54.4 (40.7-67.5) [74] | 36.9 (24.6-51.2) [40] | 6.3 (2.9-13.3) [10] |
| Male | 48.0 (34.7-61.5) [63] | 34.6 (22.7-48.7) [42] | 16.9 (8.9-29.7) [18] |
| Age |  |  |  |
| 18-29 | 34.9 (12.4-66.9) [8] | 53.8 (23.5-81.6) [4] | 11.3 (1.6-50.8) [1] |
| 30-44 | 56.3 (36.4-74.4) [22] | 31.8 (16.3-52.7) [15] | 11.9 (4.8-26.5) [8] |
| 45-59 | 53.3 (36.2-69.6) [31] | 29.1 (16.7-45.5) [21] | 16.9 (8.0-32.4) [9] |
| 60+ | 54.0 (42.6-64.9) [76] | 36.0 (26.0-47.3) [42] | 5.7 (2.5-12.2) [10] |
| Race/ethnicity |  |  |  |
| Asian | 39.5 (17.9-66.0) [11] | 57.1 (30.9-79.9) [9] | 3.4 (0.5-21.5) [1] |
| Black | 68.0 (35.1-89.3) [9] | 22.7 (6.8-54.1) [5] | 9.3 (1.8-36.2) [2] |
| Latine | 48.9 (30.4-67.8) [31] | 33.4 (17.4-54.4) [21] | 17.1 (7.1-35.6) [8] |
| White | 51.8 (40.2-63.3) [80] | 33.4 (23.6-44.9) [45] | 11.7 (6.3-20.5) [17] |
| Other/multi-race | 79.1 (33.2-96.7) [6] | 20.9 (3.3-66.8) [2] | 0 |
| Household income |  |  |  |
| Less than $50,000 | 36.2 (19.7-56.8) [38] | 40.8 (22.5-62.1) [20] | 23.0 (8.7-48.4) [8] |
| $50,000 to $99,999 | 54.5 (34.4-73.2) [42] | 40.5 (22.0-62.0) [25] | 4.0 (1.6-9.6) [7] |
| $100,000 to $149,999 | 60.3 (39.5-77.9) [26] | 32.7 (16.2-54.9) [13] | 5.8 (1.6-18.6) [3] |
| $150,000 or more | 52.2 (36.3-67.7) [31] | 33.1 (19.9-49.6) [24] | 12.4 (6.0-23.7) [10] |
| Gun ownership status |  |  |  |
| Gun owner | 54.4 (34.0-73.4) [23] | 29.9 (16.3-48.5) [17] | 14.1 (5.1-33.3) [5] |
| Non-owner who lives with owner | 72.0 (43.6-89.6) [13] | 24.0 (8.1-52.9) [8] | 4.0 (0.8-17.1) [2] |
| No guns in home | 48.9 (37.4-60.5) [98] | 38.2 (27.2-50.6) [54] | 11.2 (5.9-20.2) [20] |
| LGBT+ |  |  |  |
| Yes | 39.8 (11.1-77.7) [8] | 52.3 (17.5-85.0) [9] | 8.0 (1.7-29.7) [3] |
| No (Heterosexual, cisgender) | 49.6 (39.3-60.0) [109] | 36.9 (27.2-47.8) [68] | 12.9 (7.4-21.4) [22] |
| Veteran |  |  |  |
| Yes | 52.0 (31.0-72.4) [16] | 35.8 (18.3-58.1) [9] | 12.2 (2.9-39.4) [3] |
| No | 50.0 (39.3-60.8) [104] | 37.4 (27.4-48.7) [69] | 12.0 (6.9-20.2) [22] |

Notes: Row percentages may not sum to 100% because refusals are not shown.

# Appendix Table 13. Importance of knowing that police would be sent to the scene in cases of imminent risk, by sociodemographic characteristics (n=250)

|  | **Very important**  % (95% CI) [n] | **Somewhat important**  % (95% CI) [n] | **Not at all important**  % (95% CI) [n] |
| --- | --- | --- | --- |
| Sex |  |  |  |
| Female | 47.4 (34.2-60.9) [69] | 34.3 (22.9-47.9) [48] | 15.4 (6.3-33.2) [6] |
| Male | 54.5 (40.5-67.7) [70] | 34.5 (22.2-49.3) [42] | 10.4 (4.1-23.7) [10] |
| Age |  |  |  |
| 18-29 | 24.1 (7.4-55.9) [7] | 49.6 (20.3-79.2) [4] | 26.4 (6.8-63.9) [2] |
| 30-44 | 53.9 (33.9-72.7) [24] | 38.7 (21.6-59.2) [19] | 7.4 (1.1-36.3) [2] |
| 45-59 | 51.0 (33.8-67.8) [33] | 26.6 (15.1-42.4) [22] | 22.1 (9.6-43.3) [6] |
| 60+ | 62.5 (51.1-72.6) [75] | 29.4 (20.6-40.1) [45] | 2.1 (0.8-5.4) [6] |
| Race/ethnicity |  |  |  |
| Asian | 46.4 (22.4-72.3) [13] | 36.3 (14.3-66.1) [5] | 17.3 (3.9-51.8) [3] |
| Black | 77.0 (46.4-92.8) [10] | 23.0 (7.2-53.6) [6] | 0 |
| Latine | 50.6 (31.8-69.2) [35] | 33.0 (17.6-53.3) [23] | 16.4 (5.3-40.6) [3] |
| White | 56.1 (44.2-67.4) [79] | 28.8 (20.4-38.9) [51] | 10.7 (3.7-27.2) [9] |
| Other/multi-race | 4.5 (0.8-22.4) [2] | 85.8 (50.1-97.3) [5] | 9.7 (1.2-49.8) [1] |
| Household income |  |  |  |
| Less than $50,000 | 58.7 (37.2-77.3) [36] | 27.3 (13.5-47.6) [23] | 11.4 (2.1-43.9) [5] |
| $50,000 to $99,999 | 52.7 (32.7-71.9) [46] | 45.8 (26.5-66.3) [26] | 1.0 (0.2-4.0) [2] |
| $100,000 to $149,999 | 60.6 (39.8-78.1) [25] | 35.3 (18.5-56.8) [16] | 2.9 (0.4-18.0) [1] |
| $150,000 or more | 42.1 (27.4-58.3) [32] | 31.9 (19.4-47.7) [25] | 23.7 (11.4-42.8) [8] |
| Gun ownership status |  |  |  |
| Gun owner | 46.2 (27.0-66.5) [27] | 46.2 (25.8-68.0) [16] | 5.4 (0.8-30.1) [1] |
| Non-owner who lives with owner | 33.9 (14.4-61.0) [13] | 43.2 (17.4-73.4) [8] | 22.9 (4.0-68.1) [2] |
| No guns in home | 55.4 (43.5-66.8) [98] | 29.9 (20.4-41.6) [61] | 12.6 (5.6-25.6) [12] |
| LGBT+ |  |  |  |
| Yes | 31.1 (10.2-64.2) [11] | 34.9 (8.8-74.9) [6] | 34.0 (6.3-79.8) [3] |
| No (Heterosexual, cisgender) | 52.6 (42.0-63.0) [111] | 36.8 (27.1-47.7) [75] | 9.6 (4.3-19.8) [11] |
| Veteran |  |  |  |
| Yes | 56.9 (34.8-76.5) [17] | 34.1 (17.0-56.6) [10] | 9.1 (1.3-42.7) [1] |
| No | 52.2 (41.3-62.9) [109] | 35.4 (25.6-46.5) [71] | 11.4 (5.3-23.0) [13] |

Notes: Row percentages may not sum to 100% because refusals are not shown.

# Appendix Table 14. Importance of knowing that the person in crisis would be able to receive support in a language other than English or Spanish, by sociodemographic characteristics (n=250)

|  | **Very important**  % (95% CI) [n] | **Somewhat important**  % (95% CI) [n] | **Not at all important**  % (95% CI) [n] |
| --- | --- | --- | --- |
| Sex |  |  |  |
| Female | 60.7 (47.4-72.5) [69] | 24.6 (15.7-36.5) [39] | 12.0 (6.0-22.7) [16] |
| Male | 58.2 (44.5-70.6) [64] | 28.5 (17.9-42.2) [43] | 12.8 (6.7-23.2) [16] |
| Age |  |  |  |
| 18-29 | 77.9 (41.9-94.5) [11] | 11.3 (1.6-50.8) [1] | 10.8 (1.5-49.4) [1] |
| 30-44 | 59.7 (38.9-77.5) [25] | 30.2 (14.5-52.4) [16] | 10.1 (3.3-27.1) [4] |
| 45-59 | 57.7 (41.1-72.8) [27] | 28.8 (17.2-44.1) [25] | 13.4 (6.2-26.7) [10] |
| 60+ | 50.7 (39.5-61.7) [70] | 29.2 (20.5-39.8) [40] | 14.4 (8.0-24.7) [17] |
| Race/ethnicity |  |  |  |
| Asian | 59.5 (32.2-82.0) [12] | 23.5 (7.5-53.8) [6] | 17.0 (5.0-44.5) [3] |
| Black | 82.4 (51.6-95.4) [13] | 13.1 (2.8-44.0) [2] | 4.5 (5.6-28.3) [1] |
| Latine | 66.1 (47.5-80.7) [31] | 26.1 (14.0-43.3) [25] | 7.9 (2.0-26.7) [5] |
| White | 53.0 (41.4-64.2) [72] | 26.6 (18.7-36.3) [47] | 16.5 (10.0-26.1) [22] |
| Other/multi-race | 49.9 (12.7-87.2) [5] | 46.9 (10.8-86.6) [2] | 3.2 (0.4-23.1) [1] |
| Household income |  |  |  |
| Less than $50,000 | 65.2 (44.0-81.7) [40] | 30.0 (14.7-51.6) [23] | 2.7 (0.4-15.6) [2] |
| $50,000 to $99,999 | 59.3 (39.5-76.4) [43] | 23.0 (12.1-39.4) [22] | 17.7 (6.2-41.1) [10] |
| $100,000 to $149,999 | 68.5 (50.2-82.4) [25] | 18.2 (8.6-34.5) [10] | 12.1 (4.7-27.7) [7] |
| $150,000 or more | 53.0 (37.1-68.3) [25] | 30.4 (18.1-46.3) [27] | 14.2 (7.3-26.0) [13] |
| Gun ownership status |  |  |  |
| Gun owner | 38.2 (21.1-58.8) [17] | 41.4 (21.5-64.7) [17] | 18.8 (8.9-35.4) [11] |
| Non-owner who lives with owner | 56.7 (27.0-82.2) [11] | 19.9 (7.9-42.0) [9] | 23.4 (5.5-61.8) [3] |
| No guns in home | 65.9 (54.9-75.3) [103] | 23.3 (15.6-33.4) [52] | 8.9 (4.7-16.1) [17] |
| LGBT+ |  |  |  |
| Yes | 48.4 (15.0-83.3) [12] | 27.9 (8.6-61.4) [6] | 23.7 (3.6-72.2) [2] |
| No (Heterosexual, cisgender) | 58.5 (48.2-68.2) [103] | 28.1 (19.7-38.3) [67] | 12.6 (7.8-19.6) [29] |
| Veteran |  |  |  |
| Yes | 60.0 (38.0-78.6) [17] | 21.1 (9.0-41.9) [7] | 18.9 (6.6-43.7) [4] |
| No | 59.2 (48.5-69.0) [102] | 27.5 (19.3-37.6) [66] | 12.6 (7.4-20.6) [27] |

Notes: Row percentages may not sum to 100% because refusals are not shown.
